# Bio-inspired ultra-high energy efficiency bistable electronic billboard and reader

**DOI:** 10.1038/s41467-019-09556-5

**Published:** 2019-04-05

**Authors:** Weiran Zhang, Xiaojun Wang, Yuyang Wang, Guojian Yang, Chang Gu, Wenxuan Zheng, Yu-Mo Zhang, Minjie Li, Sean Xiao-An Zhang

**Affiliations:** 1 State Key Laboratory of Supramolecular Structure and Materials, College of Chemistry, Jilin University, Changchun, 130012 China; 2 College of Chemical Engineering and New Energy Materials, Zhuhai College of Jilin University, Zhuhai, 519041 China; 3 Changzhou IrS Optoelectronics Technology Co., Ltd., Changzhou, 213164 China

## Abstract

Bistable display has been a long-awaited goal due to its zero energy cost when maintaining colored or colorless state and electrochromic material has been highly considered as a potential way to achieve bistable display due to its simple structure and possible manipulation. However, it is extremely challenging with insurmountable technical barriers related to traditional electrochromic mechanisms. Herein a prototype for bistable electronic billboard and reader with high energy efficiency is demonstrated with excellent bistability (decay 7% in an hour), reversibility (10^4^ cycles), coloration efficiency (430 cm^2 ^C^−1^) and very short voltage stimulation time (2 ms) for color switching, which greatly outperforms current products. This is achieved by stabilization of redox molecule via intermolecular ion transfer to the supramolecular bonded colorant and further stabilization of the electrochromic molecules in semi-solid media. This promising approach for ultra-energy-efficient display will promote the development of switching molecules, devices and applications in various fields of driving/navigation/industry as display to save energy.

## Abstract (web summary)

For electrochromic materials to reach their full potential for high efficiency bistable displays, technical challenges related to their underlying mechanism must be addressed. Here, the authors, through intelligent molecular design, report a solid bistable device with state-of-the-art performance.

## Introduction

Although steady progress has been made on improving power efficiency^1,2^, nearly 50% electricity is still used for display in many consumer electronics^3^, which consume over 10% of office and residential electricity. Developing bistable media for energy-efficient displays has proved to be extremely challenging, but very important for global energy-saving and sustainability. Although pioneer of bistable display, such as e-ink, has made steady progresses^4,5^, its intrinsic weakness of lower reflectance and contrast ratio still exists. Meanwhile, though long-anticipated electrochromic (EC) materials for bistable display have promising results^6–12^ and favorable attributes as prints on paper^13,14^, various shortcomings including slow color-switching rate, short color-duration, poor reversibility, and limited color variations and purity remain unresolved, which greatly restrict their practical applications. Aforementioned problems (e.g., e-ink’s electrophoretic charge-repelling, unstable change of redox-colorants’ energy states of traditional EC materials) are solely related to their mechanisms. For the e-ink system, electrophoretic method is used to move charged particles/microcapsules back and forth to display the color information. However, due to the drawbacks of charges repulsion, highly viscous media and frequent voltage retreatment have to be used for increasing its bistability. These problems insurmountably lead to slow coloration switching time and higher power consumption. In addition, the light scattering and refraction on these microcapsules result in undesirable poor color purity and color concentration. For traditional EC materials, the electrochromism relies on change of redox states of the EC molecules. During the coloration process, their reduction/oxidation leads to the color change of the EC materials directly. Unstable radical always appears in either reduction or oxidation state, which may cause device degradation and unsatisfactory electrochromic properties under such unstable energy state. We have tried to overcome these drawbacks through some different mechanisms such as bond-coupled electron transfer (BCET)^15^ and realized excellent electrochromic properties; however, the stability of the device is to be improved for limited long-term stability of related molecules.

Fortunately, we discovered that these problems can be avoided by disconnecting the coloration moiety from redox-changing subunit (using coenzyme-Q-like redox-active molecule as electrobase), which can induce pH change electrically to switch pH-sensitive dyes^16–20^. Thus, charge repulsion, unstable radical, and/or higher energy state of redox-dyes associated with conventional electrochromic/e-ink pathways were avoided. This process accompanied with proton-coupled electron transfer (PCET) broadens color selection with available pH dyes and improves redox species’ stability by avoiding reactive radicals. Advantages of such biomimetic chemistry enable fine tuning of these properties by molecular structure design and modification. However, its bistability and switching speed were still unsatisfactory to an ideal goal.

Here, supramolecular interactions between suitable electroacid and dyes as colorants are also essential, which act as supramolecular-glue, to closely associate them together. This not only stabilizes redox molecule via facilitated PCET and structure alterations among them with dyes, but also, stabilizes switched-dyes further with supramolecular cohesion and dynamic interactions among surrounded electrolyte charge transfer chains. Finally, we investigated whether we can really demonstrate the feasibility of making a simple device with all properties for ideal bistable display.

## Results

### Feasibility

**Fig. 1:**
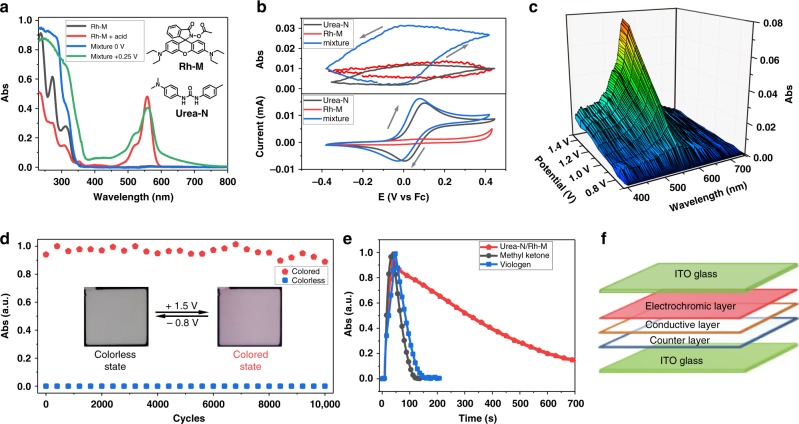
Feasibility and device properties of the “**Urea-N** +** Rh-M**” electrochromic system. **a** UV-vis spectra of **Rh-M** (black, 1.0 × 10^−4^ mol L^−1^), **Rh-M** (1.0 × 10^−5^ mol L^−1^) treated with CF_3_COOH (red), “**Urea-N** + **Rh-M**” (blue, **Rh-M**: 1.0 × 10^−4^ mol L^−1^, **Urea-N**: 1.0 × 10^−4^ mol L^−1^), and “**Urea-N** + **Rh-M**” under + 0.25 V vs. Ag/AgNO_3_ (green, **Rh-M**: 1.0 × 10^−4^ mol L^−1^, **Urea-N**: 1.0 × 10^−4^ mol L^−1^). **b** Changes in absorption at 560 nm (top) during CV (bottom) of **Urea-N** (1.0 × 10^−3^ mol L^−1^), **Rh-M** (1.0 × 10^−3^ mol L^−1^) and “**Urea-N** **+** **Rh-M**” (1.0 × 10^−3^ mol L^−1^/1.0 × 10^−3^ mol L^−1^) in acetonitrile at 20 mV s^−1^. **c** 3-D spectroelectrochemical diagram of “**Urea-N** + **Rh-M**” solution at +0.8 V–+1.5 V for 15 s in liquid device. **d** The stability at 560 nm of the “**Urea-N** + **Rh-M**” liquid device (10000 cycles). **e** The optical memory of liquid devices of “**Urea-N** + **Rh-M**” (red), methyl ketone (black), and viologen (blue) at 560 nm after the electrochromic stimuli are cut off. **f** Structure of the semi-solid bistable electrochromic device

1-(4-(dimethyl-amino)phenyl)-3-(p-tolyl)urea (**Urea-N**, see Supplementary Figs. 1, 46, 47) was selected as an ideal electroacid due to its ability of proton release after the reversible two-electron oxidation as a urea derivative^21^ to stimulate the pH-sensitive rhodamine B derivative 3′,6′-bis(diethylamino)-3-oxospiro[isoindoline-1,9′-xanthen]-2 yl-acetate (**Rh-M**, see Supplementary Figs. 2, 3, 48, 49 and Supplementary Table 1)^22^ to realize electrochromism. In order to test the feasibility of the electrochromism, the in situ UV-vis and fluorescence spectroscopy of the mixture and individual solutions were tested accordingly. As shown in Fig. 1a and Supplementary Fig. 4, the solution with a mixture of **Urea-N** and **Rh-M** shows new absorption (560 nm) and fluorescence (583 nm) peak, which is similar to the peak of **Rh-M** stimulated by chemical acid CF_3_COOH, while the individual solutions have no obvious change in either absorption or fluorescence spectra under positive voltage (+0.25 V vs. Ag/AgNO_3_). The cyclic voltammetry with in situ UV-vis and fluorescence spectroscopy of the solutions were also monitored. The absorption of **Rh-M** (560 nm) is able to be regulated by electrical field only when **Urea-N** and **Rh-M** coexist in solution, and the intensity of absorption at 560 nm is dependent on the redox state of **Urea-N** (Fig. 1b). The intensity of absorption begins to increase when **Urea-N** is oxidized at −0.1 V vs. Fc, and decreases right after the potential is switched back to the reductive peak of **Urea-N**, and finally vanishes. The fluorescence spectra indicate the similar tendency to the absorption spectra (Supplementary Fig. 4f). These experiments above undoubtedly demonstrate that **Rh-M** is stimulated by the proton released from **Urea-N**^21^ when oxidized under positive voltages to realize the absorption and fluorescence change. When applied negative voltages, the oxidized **Urea-N** was reduced and the proton transferred inversely to realize a whole reversible electrochromic process. To investigate the photo stability and thermal stability of the **Urea-N** and **Rh-M**, which will further guarantee their possibility as electrochromic materials, the absorption spectra of **Urea-N** and **Rh-M** on ring-open/close form in dark/under sunlight and under different temperatures were also tested and showed no obvious change with/without light or under different temperature from 25 ^o^C to 75 ^o^C (Supplementary Figs. 34–36). Therefore, their electrochromic properties will not be affected by light or temperature to some extent. Till now, the feasibility of electrochromism has been proved by the above tests, and the electrochromic mechanism is discussed in the next section.

The simple liquid device which consists of EC materials (**Urea-N** and **Rh-M**), electrolyte (1-butyl-3-methylimidazolium hexafluorophosphate, [BMIM]PF_6_), and p-benzoquinone (BQ) as counter compound was fabricated right after the feasibility of electrochromism was proved to test the electrochromic properties of the electroacid system in device (Supplementary Fig. 5). The switchability of the liquid electrochromic device was tested first, and spectra showed that the intensity of absorption (Fig. 1c) and fluorescence (Supplementary Fig. 5c) start to increase under + 0.8 V. Other EC properties of the liquid device were also measured, such as coloration efficiency, stability, and optical memory. To our delight, the device exhibits superior stability that can be stimulated reversibly over 10,000 times with no obvious degradation (Fig. 1d; Supplementary Fig. 6). Another impressive result shows that the device displays much longer optical memory than other traditional EC materials under same condition (Fig. 1e), such as viologen and methyl ketone^16^, and can even maintain the colored state for 7 days in the liquid device when the solution was isolated by a proton transfer membrane (Supplementary Fig. 7). The excellent optical memory of the liquid device provides the possibility of realizing bistable electrochromic display with our electroacid mechanism.

### Bistable device

**Fig. 2:**
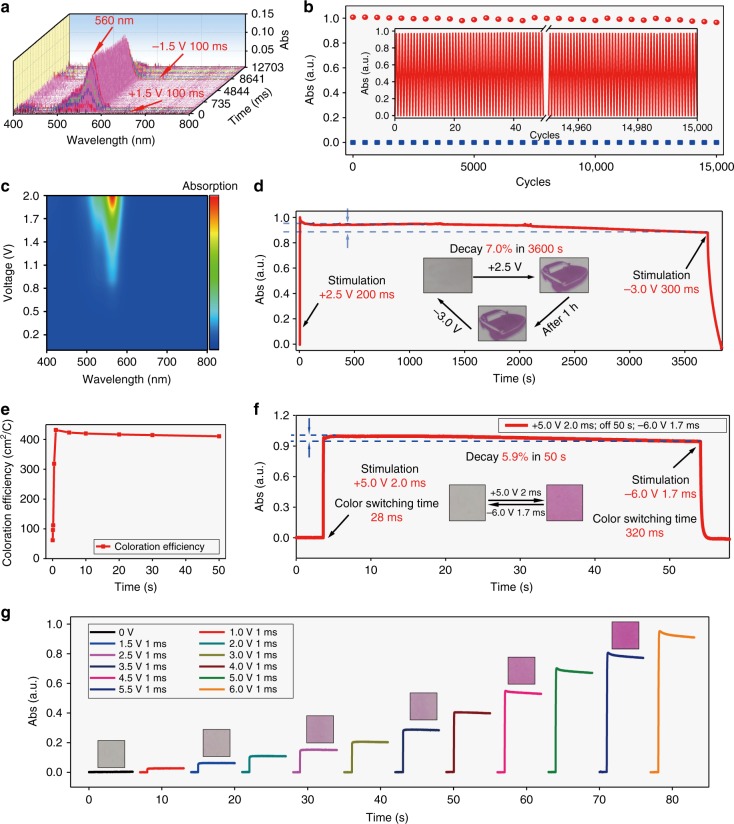
Electrochromic properties of solid devices. **a** Their absorption from 400 to 800 nm measured by fiber optical spectrometry under +1.5 V 100 ms, power off 8 s, −1.5 V 100 ms. **b** The stability of the device under stimulation of +1.0 V 0.6 s, wait 1.0 s, −0.5 V 1.4 s, wait 3.0 s. **c** The absorption spectra of the device under different voltages for 200 ms. **d** The absorption at 560 nm under stimulation of +2.5 V 200 ms, power off 3600 s, −3.0 V 300 ms. Inset: pictures of the bistable device. **e** Coloration efficiency of the device. **f** The absorption of solid Li–Nafion bistable device at 560 nm under +5.0 V 2.0 ms, power off 50 s, −6.0 V 1.7 ms. **g** Absorption spectra of the Li–Nafion device at 560 nm under different voltages for 1 ms. Inset: pictures of the Li–Nafion device under different voltages

In order to further realize bistable electrochromic device upon electroacid mechanism, the semi-solid device was fabricated by embedding the above materials in poly(methyl methacrylate) (PMMA) and constructing layered structure as shown in Fig. 1f. Apart from the electrochromic materials (**Urea-N** as electroacid and **Rh-M** as pH sensitive molecule), BQ was also added to the electrochromic layer of the solid device to help capture the proton to speed up the color-fading process under negative voltages due to its electrobase properties^17–19^ (Supplementary Note 4 and Supplementary Figs. 27–30). In order to test the electrochromic properties of solid devices, the absorption spectra from 400 to 800 nm of the device were measured first by fiber optical spectrometry, as shown in Fig. 2a. After a stimulation of +1.5 V 100 ms, the power was cut off, and the absorption intensity showed no degradation before the negative voltage (−1.5 V 100 ms) was applied, which means the solid device exhibits highly anticipated bistable property. After the optimum parameters for bistable solid devices were obtained (Supplementary Note 3, Supplementary Fig. 9 and Supplementary Table 3), the bistability of the device was also observed and it was delightful to discover that the device remained >93% of its absorption after each switch for more than 1 h (Fig. 2d) and possessed superior stability of 15,000 cycles with no degradation (Fig. 2b), even more than 10,000 cycles with bistability (Supplementary Fig. 10), which is the best known solid bistable electrochromic device with superb stability as far as we know. The solid devices also show low switch-on voltage of +0.8 V and unique color gradient by changing stimulation voltages or time, which makes the important display’s grayscale possible (Fig. 2c; Supplementary Fig. 11). The device’s coloration efficiency is 430 cm^2^ C^−1^ (Fig. 2e), which is, another important character, good enough for display, and higher than most known electrochromic materials. In addition, such devices exhibit much shorter voltage stimulation time for coloration (2.0 ms, +5.0 V) or decoloration (color-off state) (1.7 ms, −6.0 V) with slightly delayed completion of coloring or color fading (28 or 320 ms) (see Supplementary Fig. 15), goodish bistability (Fig. 2f; Supplementary Fig. 16) and color gradient properties (Fig. 2g; Supplementary Fig. 14) when using lithiated Nafion-212 (Li–Nafion) membrane as conductive layer (Supplementary Fig. 12), which are ten times faster than current e-windows and e-billboards.

**Fig. 3:**
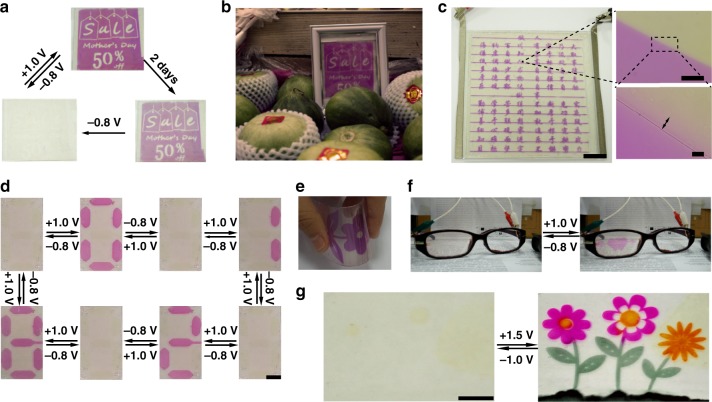
Potential applications of bistable solid devices. **a** The e-billboard prototype (10 cm × 10 cm) performance. **b** The prototype for supermarket e-billboard/reader. **c** The prototype for high-resolution e-reader/display, the scale bars are 2 cm, 200 μm, 20 μm, respectively. **d** The prototype for pixel e-billboard/reader, scale bar: 1 cm. **e** The prototype for flexible display. **f** The prototype for wearable thin-film display on eye-glasses. **g** The prototype for reversible multicolor display, scale bar: 1 cm

We gladly discover that such device is far superior for long-lasting information display (energy-saving is >99% than e-ink reader and related LCD/LED devices when continuous display for 2 days). Take the information display in price-card-prototype for supermarket e-billboard/reader (10 cm × 10 cm) for example (Fig. 3a, b), it can be clearly displayed for 2 days with <1.0 J of energy consumption even applied an auxiliary voltage to make the display much more clear every half-day, but our display only consumes under one ten-thousandth of energy in comparison with conventional LCD and LED e-billboard/readers for 2 days information display^23–25^ (Supplementary Note 5). This price-card-prototype is easily applicable in market to show colorful electronic display. Ultra-high resolution is another characteristic of this system. Detailed examination of our device’s coloration image (Fig. 3c) under microscope reveals that its diffusion length is less than 20 μm and the sample can be identified clearly in 40 μm × 40 μm dot. This reflects that device has ultra-high photographs quality resolution of 635 PPI (pixels per inch) with this material theoretically, which is much higher than current technologies for bistable display including e-ink^23–25^. Flexibility and wearability are two long-awaited features for future display and are demonstrated hereon (Fig. 3e; Supplementary Figs. 38, 39 and Supplementary Movie 5). No disruption of performance can be observed even though such device was arbitrarily bended ≥85^o^ angle, and robust enough to remain its electrochromic properties after 300 bending/de-bending cycles test (Supplementary Fig. 40). This indicates that such device is extremely flexible and might be suitable for ideal paper-like display. A prototype of wearable display (Fig. 3f; Supplementary Figs. 41, 42, and Supplementary Movie 6) was also constructed accordingly with our materials. Different patterns and letters can be exhibited in related solid devices (Supplementary Fig. 37), and pixel display was also realized to achieve potential dynamic display (Fig. 3d, Supplementary Movies 1–4). When pH-sensitive dyes with different colors were used together in the device, multi-color devices were also achieved by mono-layered pixilated-device (Fig. 3g; Supplementary Figs. 43,  44 and Supplementary Table 4) or multi-layered stacked electrodes^20^. Information can be displayed, such as pictures, numbers, words (see Fig. 3d; Supplementary Fig. 37, and Supplementary Movies 1, 5, 6), with a single switch at +1.0 V, and maintained with zero power consumption, and be erased/rewritten repeatedly with −0.8 V/ +1.0 V. In addition, ingredients used in our media are proved to be very safe by cytotoxicity-tests on cells with MTT (3-(4,5-dimethylthiazol-2-yl)−2,5-diphenyltetrazolium bromide) assays (Supplementary Fig. 45).

### Mechanism of such bistable electrochromic device

**Fig. 4:**
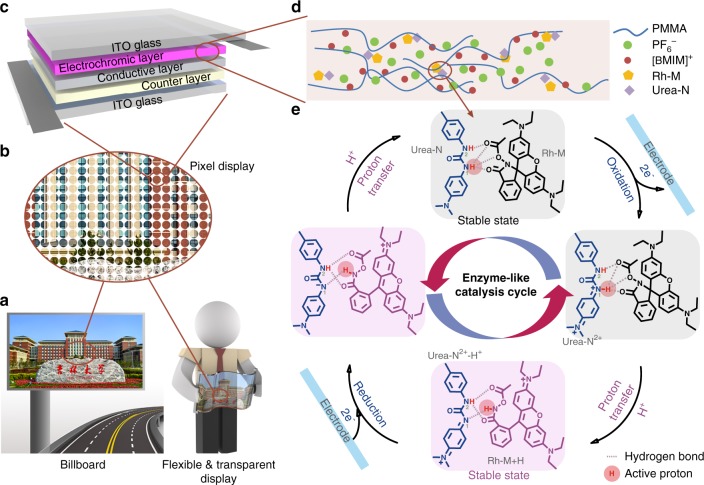
Schematic diagrams and mechanism of the “**Urea-N** + **Rh-M**” electrochromic system. **a** Schematic of energy-saving billboard (left) and flexible transparent display (right). **b** Schematic of pixel display. **c** Structure of bistable electrochromic device. **d** The composition of the electrochromic layer. **e** The proposed mechanism (gray dashed line: hydrogen bonding between **Urea-N** and **Rh-M**; red circle: active proton that transferred between **Urea-N** and **Rh-M**)

These properties above of this unique bistable electrochromic devices are superior to known EC devices, which endow them good potential for future displays. The reaction mechanism (Fig. 4e) to achieve such extraordinary results is further summarized as following:

First, the bonded **Urea-N** and **Rh-M** (H-complex), via hydrogen-bonding and other supramolecular forces has facilitated the PCET process, which leads to the stable redox intermediates, and high reversibility. The oxidation state of H-complex is very stable, once the ionic diffusions/exchanges between working and counter electrodes are eliminated in the device. The interactions between **Urea-N** and **Rh-M** have been proven with several experiments, such as ^1^H-NMR titration (Supplementary Figs. 17–20), cyclic voltammogram (Supplementary Fig. 21), and DFT analyses^26^ (Supplementary Figs. 22–23). As the most widely used strategy to demonstrate hydrogen bonding, ^1^H-NMR titration has been tested between **Urea-N** and **Rh-M** compared with other control molecules with specially modified functional groups. According to Supplementary Figs. 17–20, both H on this urea moiety shift to lower field, which indicates the interaction between the **Urea-N** and **Rh-M**, but meanwhile, there is no shift in mixture of **Urea-N** and the control molecules. Cyclic voltammetry of **Urea-N** with different equivalent of **Rh-M** was observed then, as shown in Supplementary Fig. 21, the reversible oxidative peak of **Urea-N** increased further in height, and shifted to the low potential when the concentration of **Rh-M** increased. To clearly figure out the proton transfer route of the electroacid mechanism, the potentials of both “**Urea-N**^2+^ with **Urea-N**” and “**Urea-N**^2+^ with **Rh-M**” H-bond complex were calculated accordingly though DFT theory (M06/6–311 g(d,p)) to study the effect of intermolecular ion transfer on the redox molecule, Supplementary Figs. 22, 23 herein show the two possible routes of intermolecular proton transfer—the proton transferred either from oxidized **Urea-N** to its neutral state **Urea-N**^21^ or from oxidized **Urea-N** to its adjacent **Rh-M**. Compared with the first route, the proton transfer reaction occurs more likely in a hydrogen-bond complex between **Urea-N**^2+^ and adjacent **Rh-M** from thermodynamics perspective (ΔG = −9.9 kcal mol^−1^, exothermic reaction) and dynamics perspective (ΔE = 103.0 kcal mol^−1^, a low barrier). The lower potential energy of “**Urea-N**^2+^ + **Rh-M**” complex indicates that the intermolecular proton transfer between oxidized **Urea-N** and its adjacent **Rh-M** helps to form a more stable complex (H-complex) via hydrogen bonding, which further stabilizes the redox species (Supplementary Fig. 33). We found that such H-complex can not only stabilize its redox intermediates (ΔG = −9.9 kcal mol^−1^ compared with ΔG = 2.6 kcal mol^−1^), but also facilitate fast and efficient proton transfer (barrier is reduced by 40 kcal mol^−1^). To better understand the effect of supramolecular interaction between **Urea-N** and **Rh-M** through diffusion ability of the dynamic complex, the DOSY (diffusion-ordered spectroscopy) of **Urea-N** with different ratio of **Rh-M** is tested (Supplementary Fig. 31), accordingly. Despite the relatively high strength of hydrogen bonding within H-complex, no obvious diffusion restriction caused by hydrogen bonding can be detected somehow (Supplementary Fig. 31 and Supplementary Table 2). This seems to indicate that the redox molecule **Urea-N** might have uneasily changeable diffusion ability to some extent. This unusual phenomenon has similarity somehow with the long-puzzling ultrafast signal conduction property of nerve system/nerve cells. Wherein, their protein-networks are formed by numerous protein molecules and others. These molecular aggregates are interconnected and stabilized by hydrogen bonding and other supramolecular forces, and having ultra-active dynamic alterations and enzymatic functions with negligible diffusion rates. Therefore, the role of **Urea-N** here resembles the “redox-enzyme”, which interacts with adjacent **Rh-M**, and donates proton to induce **Rh-M**’s structural tautomerization via highly reversible redox (Fig. 4e). The supramolecular interactions between **Urea-N** and **Rh-M**, especially hydrogen bonding not only stabilize the complex in both colorless and colored states to facilitate the bistable properties, but also accelerate the fast and efficient PCET process.

The hydrogen-bonding and supramolecular interactions can be further proved by experiments with control molecule 3-(4-(dimethylamino)phenyl)-1-methyl-1-(p-tolyl)urea  (**Urea-Me**, see Supplementary Figs. 25a, 50, 51), in which the H atom on N(2) is substituted by methyl compared with **Urea-N**. As shown in Supplementary Fig. 25b, when mixed with **Rh-M**, the chemical shift of H on N(1) in **Urea-Me** has no change, on the contrary, both H of urea moiety in **Urea-N** shift obviously (Supplementary Figs. 17, 28), which indicates the hydrogen bond between **Urea-N** and **Rh-M** is much stronger. According to Supplementary Fig. 25c, **Urea-Me** can also be oxidized under positive voltage while **Rh-M** has no redox reaction in that range, which provides the possibility of **Urea-Me** being an electroacid to switch **Rh-M** to make color change. The electrochromic mechanism of **Urea-Me** as electroacid is proposed in Supplementary Fig. 26. Unlike **Urea-N**, after being oxidized and release proton, the control molecule **Urea-Me** is not able to provide an additional H atom to form hydrogen bonding again with **Rh-M** after its oxidation and proton transfer, which leads to the different electrochromic performances of device. Compared with the UV spectrum of mixture of **Urea-Me** and **Rh-M** at 560 nm after the electrical oxidation, the spectrum of mixture of **Urea-N** and **Rh-M** shows longer optical memory and better bistability, which clearly indicates this colored state is actively stabilized by the supramolecular interaction between **Urea-N** and **Rh-M**. Meanwhile, the faster coloring response of “**Urea-N** + **Rh-M**” (larger slope of red line in Supplementary Fig. 25d) also illustrates that the proton transfer between electroacid and pH-sensitive dye can be accelerated by the intermolecular hydrogen bonding (supramolecular interaction). In addition, the comparison of the bistablity of solid devices among different EC materials also shows that the hydrogen bonding between **Urea-N** and **Rh-M** plays an important role in the bistable state (see Supplementary Note 4 and Supplementary Fig. 24).

Second, the ionically interlinked and interlaced electrolyte chains play a crucial role on the bistable property as shown in Fig. 4d. In such thin-film system, there are larger amount of electrolytes, which weakly associate with −CO_2_− atomic groups of PMMA via supramolecular interaction, to form well distributed and interlaced ionic electrolyte chains based on the reference^27^ and our related experimental results in Supplementary Fig. 32. Such PMMA-bounded dynamic-flexible [BMIM]PF_6_ semi-solid electrolyte interacts with dipolar atomic groups of the adjacent “**Urea-N** + **Rh-M**” H-complex due to their structural similarity with the planar cationic BMIM. They facilitate further the double-redox process of **Urea-N**, via intermolecular PCET, and stabilize the ionized color switch molecules and their ionized intermediates also (i.e., its oxidized **Urea-N** and this electroacid switched ring-open form of **Rh-M**) by speeding up the proton transfer via charges redistribution during redox process.

## Discussion

In summary, one of the best known solid bistable electrochromic devices with superb stability has been achieved so far with an unconventional biomimetic electrochromic mechanism. It shows superior bistable property, excellent stability, very high coloration efficiency and relatively low turn on/off (color switching) voltage, extremely high energy efficiency, and maintaining the information/display with zero energy consumption. Several promising prototypes (e.g., e-billboard, smart glasses, rewritable e-paper and reader, flexible display, and multi-color display) were successfully constructed and demonstrated. Success of this is due to introduction of switchable dyes in the specially designed and supramolecularly bounded H-complexes, which are adjacent with interlaced electrolyte chains of dynamic-flexible [BMIM]PF_6_, to improve their color switching speed and bistability via increasing the electron/proton transfer, and avoiding charge repulsion of the color substances. This unconventional biomimetic system not only facilitates the reversible double-redox-process of **Urea-N**, but also stabilizes the ionized color-switch molecules via inter-/intra-molecular electron/ion-hopping and dynamic charges stabilizations. Thus, unstable radical states of colorants are avoided to improve device’s reflectance, contrast ratio, and long-term chemical stability. This unconventional biomimetic exploration for better applications is undoubtedly a starting point, and may  lead and inspire more researches, better materials and biomimetic technologies for future display, medical detection, and other applications, along with deeper understanding nature’s way in molecular biology, cell biology, and biochemistry, etc.

## Methods

### Preparation of Urea-N, Rh-M

**1-(4-(dimethylamino)phenyl)-3-(p-tolyl)urea (Urea-N):** 1-isocyanato-4-methylbenzene (10 mmol) was dissolved in 40 mL THF, then added to a solution of 10 mmol of *N, N*-dimethylbenzene-1,4-diamine also dissolved in 40 mL THF at 4 ^o^C. The reaction was stirred for 10 h under N_2_ atmosphere. The solvent of the reaction mixture was distilled off after the reaction, and the residue was then recrystallized with ethanol, 87% yield (see Supplementary Figs. 1, 46, 47). ^1^H NMR (500 MHz, DMSO-*d*_*6*_) δ 8.38 (s, 1 H), 8.22 (s, 1 H), 7.31 (d, J = 8.3 Hz, 2 H), 7.25 (d, J = 8.9 Hz, 2 H), 7.06 (d, J = 8.3 Hz, 2 H), 6.69 (d, J = 9.0 Hz, 2 H), 2.82 (s, 6 H), 2.23 (s, 3 H). ^13^C NMR (126 MHz, DMSO-*d*_*6*_) δ 152.85, 146.41, 137.48, 130.16, 129.59, 120.10, 118.04, 113.15, 40.70, 20.30. LC-HRMS: m/z calc. for C_16_H_20_N_3_O 270.1601, found 270.1545.

**3′,6′-bis(diethylamino)-3-oxospiro[isoindoline-1,9′-xanthen]-2-yl acetate (Rh-M):** Acetyl chloride (5 mmol) was slowly added into the mixture of 3′,6′-bis(diethylamino)-2-hydroxyspiro[isoindoline-1,9′-xanthen]-3-one (1.25 mmol) and triethylamine (3.7 mmol) in 15 mL of 1,2-dichloroethane at 0 ^o^C. The reaction was stirred at room temperature for 4 h. Then the solvent of the reaction mixture was distilled off after the reaction. The crude product was purified by column chromatography to afford a white solid, 65% yield (see Supplementary Figs. 2, 48, 49). ^1^H NMR (500 MHz, CDCl_3_) δ 7.93 (d, J = 6.9 Hz, 1 H), 7.54–7.39 (m, 2 H), 7.09 (d, J = 7.2 Hz, 1 H), 6.69 (d, J = 8.9 Hz, 2 H), 6.42–6.24 (m, 4 H), 3.34 (q, J = 7.1 Hz, 8 H), 2.02 (s, 3 H), 1.16 (t, J = 7.0 Hz, 13 H). ^13^C NMR (126 MHz, CDCl_3_) δ 167.05, 163.25, 153.73, 151.03, 149.08, 133.34, 129.30, 128.46, 127.90, 123.96, 123.42, 107.97, 104.27, 97.71, 65.87, 44.46, 18.29, 12.76. LC-HRMS: m/z calc. for C_30_H_34_N_3_O_4_ 500.2544, found 500.2514.

### Preparation of the semi-solid electrochromic device

Electrochromic solution: A mixture of PMMA (3.5 g), [BMIM]PF_6_ (1.7 mL), **Urea-N** (81 mg), **Rh-M** (150 mg), and BQ (110 mg) in 20 mL of acetonitrile was stirred for 24 h. Conductive solution: a mixture of PMMA (3.5 g) and [BMIM]PF_6_ (1.7 mL) in 20 mL of acetonitrile was stirred for 24 h. Counter solution: a mixture of PMMA (3.5 g), [BMIM]PF_6_ (1.7 mL), BQ (220 mg), and hydroquinone (440 mg) in 20 mL of acetonitrile was stirred for 24 h.

First, the transparent counter film layer was deposited by spin coating on the first ITO. Next, the transparent conductive film was deposited on the top of the counter film. Second, the transparent colorless electrochromic film was deposited by spin coating on the second ITO. Finally, the two ITO are assembled together as Supplementary Fig. 8.

### Preparation of the Li–Nafion membrane

Nafion-212 ionomer film in H^+^ form was stirred in a solution of 1.0 M LiOH in the mixture of 20 mL of H_2_O and 30 mL of EtOH at 80 ^o^C for 12 h. The lithiated Nafion ionomer film was rinsed in boiling deionized water and then to remove the remaining salt and organic solvent. After vacuum drying at 120 ^o^C overnight, it was transferred into an argon-filled glove box^28^. The IR spectra are as shown in Supplementary Fig. 13.

Li–Nafion membrane was soaked into deionized water for 2 h to enhance the conductivity and the water on the surface of the membrane was then removed by filter paper before use.

## Supplementary information

Supplementary Information

Description of Additional Supplementary Files

Supplementary Movie 1

Supplementary Movie 2

Supplementary Movie 3

Supplementary Movie 4

Supplementary Movie 5

Supplementary Movie 6

## Data Availability

The authors declare that the main data supporting the findings of this study are available within the article and its Supplementary Information files.
