# Supplementary material for: Bio-inspired ultra-high energy efficiency bistable electronic billboard and reader
Source: Nat Commun. 2019 Apr 5;10:1559. doi: 10.1038/s41467-019-09556-5 (PMC6450890; doi:10.1038/s41467-019-09556-5)
Supplement: Supplementary file 1 — Supplementary Information [file 41467_2019_9556_MOESM1_ESM.pdf]

Supplementary Information

Bio-inspired Ultra-high Energy Efficiency Bistable Electronic Billboard and Reader

Zhang *et al.*

Supplementary Figures

Supplementary Figures 1 to 51

Supplementary Tables

Supplementary Tables 1 to 4

Supplementary Notes

Supplementary Note 1: Light and thermal stability of Urea-N and Rh-M
Supplementary Note 2: Electrofluorochromic performance in liquid devices
Supplementary Note 3: Optimum parameters of the solid device
Supplementary Note 4: The electrochromic mechanism
Supplementary Note 5: Calculation of energy consumption of the bistable billboard

Supplementary Methods

Supplementary Method 1: Materials and Methods
Supplementary Method 2: Synthesis of molecules
Supplementary Method 3: Preparation of the electrochromic devices

Supplementary References

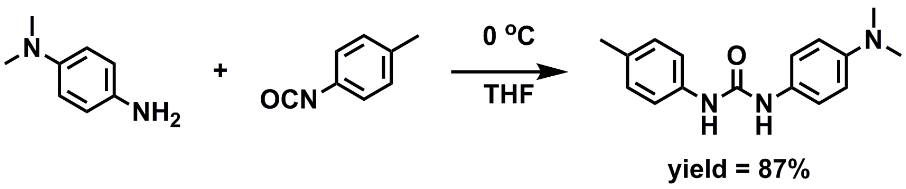

Supplementary Figure 1. Synthetic route of Urea-N.

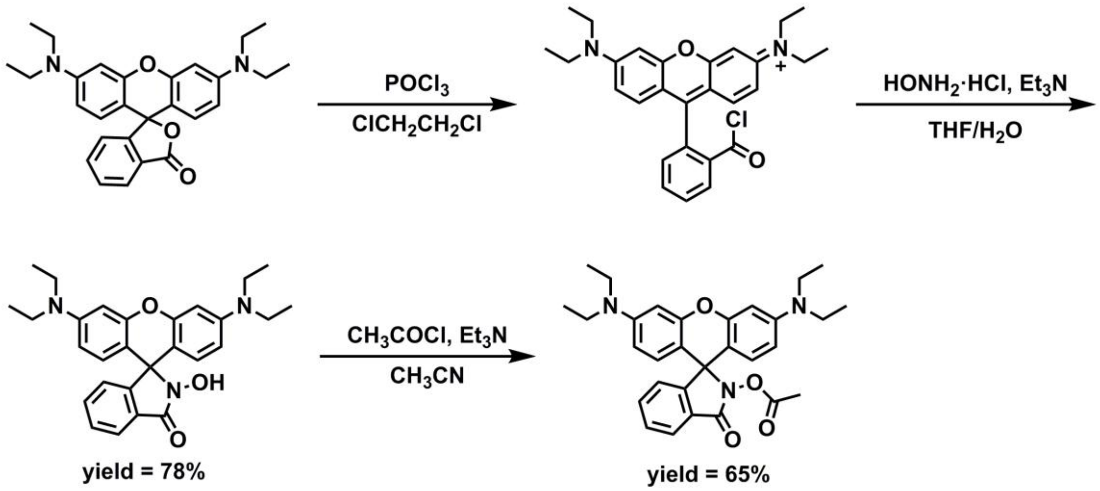

Supplementary Figure 2. Synthetic route of Rh-M.

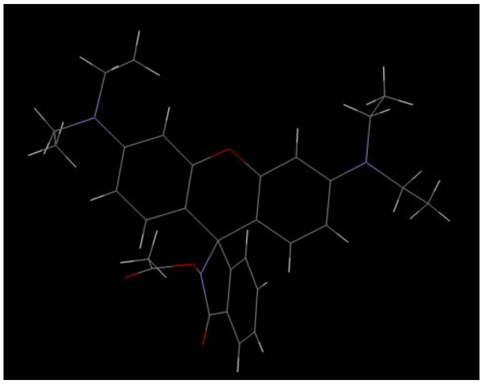

Supplementary Figure 3. Crystal structure of Rh-M.

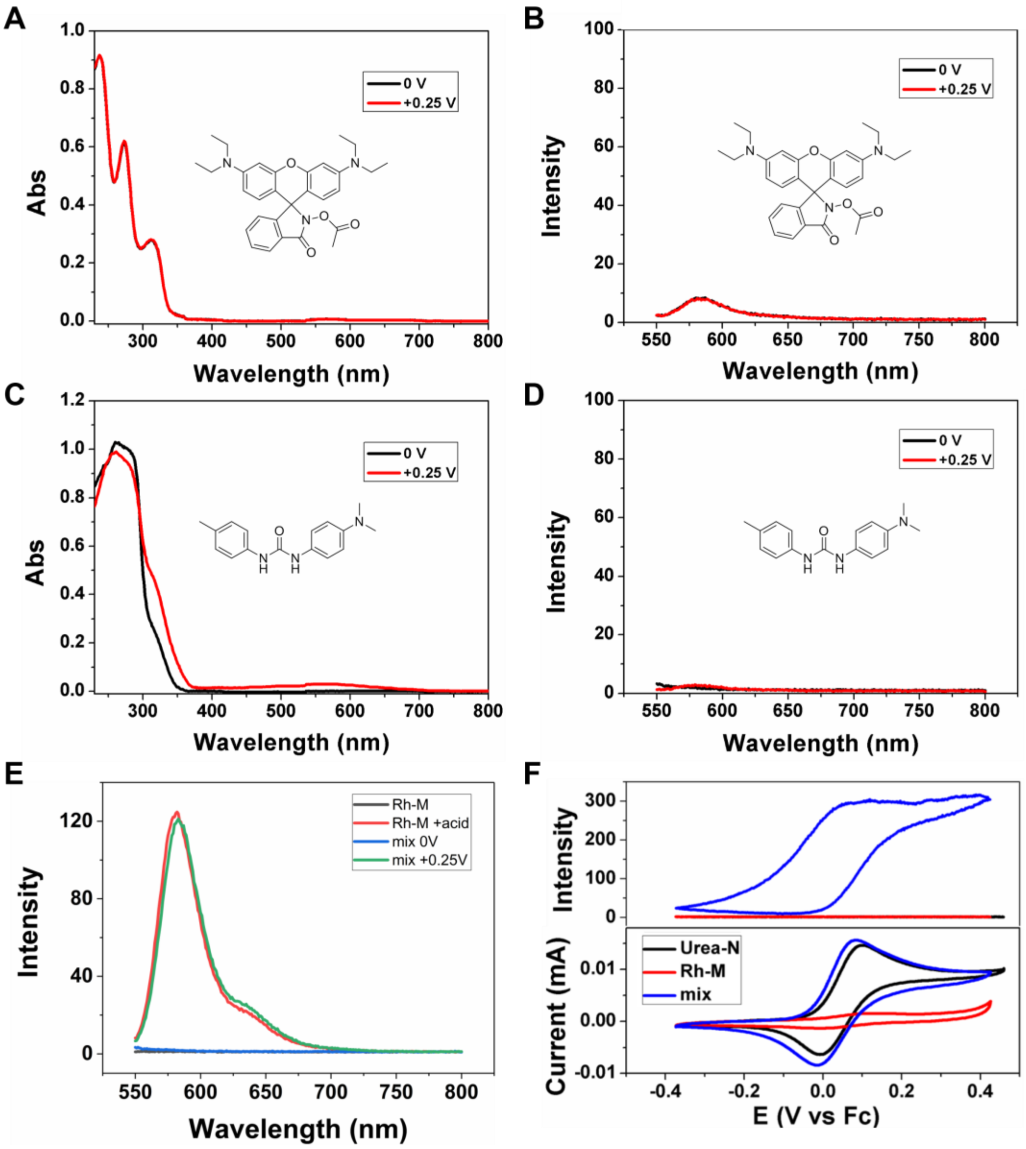

Supplementary Figure 4. The feasibility of electrochromism and electrofluorochromism. The absorption spectra and the fluorescence spectra of Rh-M (1.0×10^{-4} mol L^{-1}, A, B) and Urea-N (1.0×10^{-4} mol L^{-1}, C, D) solution at 0 V (black), at +0.25 V vs Ag/$AgNO_3$ (red), (E) fluorescence spectra of Rh-M (black, 1.0×10^{-4} mol L^{-1}), Rh-M (1.0×10^{-5} mol L^{-1}) treated with CF_3COOH (red), Urea-N/Rh-M (blue, Rh-M: 1.0×10^{-4} mol L^{-1}, Urea-N: 1.0×10^{-4} mol L^{-1}), and Urea-N/Rh-M under +0.25 V (green, Rh-M: 1.0×10^{-4} mol L^{-1}, Urea-N: 1.0×10^{-4} mol L^{-1}); (F) Changes in fluorescent intensity at 583 nm (top) during CV (bottom) of Urea-N (1.0×10^{-3} mol L^{-1}), Rh-M (1.0×10^{-3} mol L^{-1}) and Urea-N/Rh-M (1.0×10^{-3} mol L^{-1}/1.0×10^{-3} mol L^{-1}) in acetonitrile at 20 mV s^{-1}.

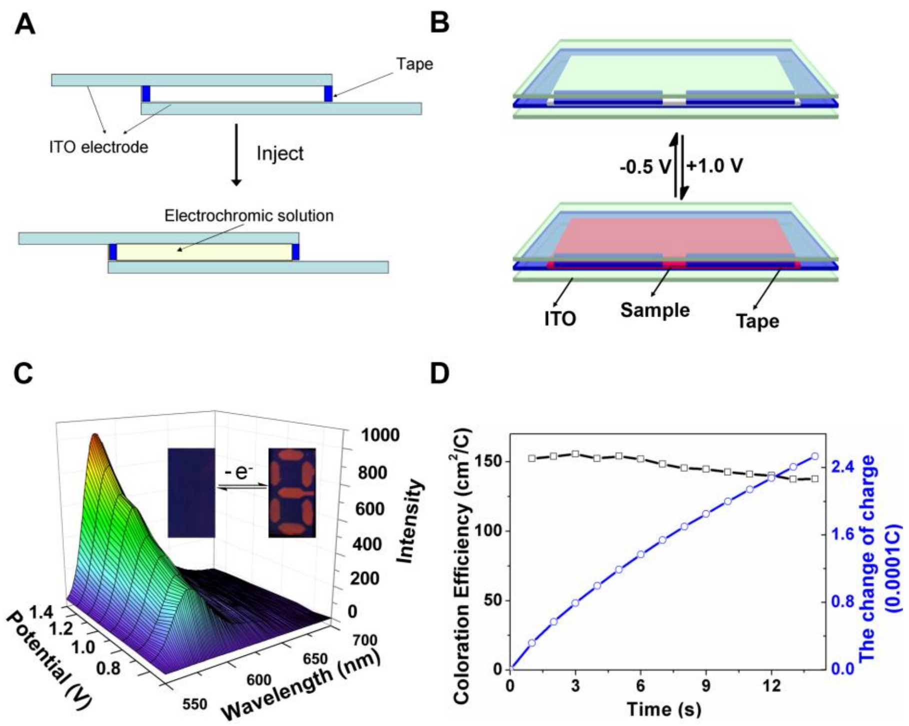

Supplementary Figure 5. The structure and performance of liquid device. (A) The outline of the preparation of liquid electrochromic device. (B) Schematic diagram of electrochromic process liquid thin film devices. (C) 3-D spectroelectrochemical diagram of Urea-N/Rh-M mixture solution at +0.8 V - +1.5 V for 15 s in liquid device. Inset: Photographs of liquid device on emissive mode (ex: 365 nm) under +1.0 V using "8" pattern. (D) Coloration efficiency (black) and the charge change (blue) as a function of switch time for Urea-N/Rh-M liquid device.

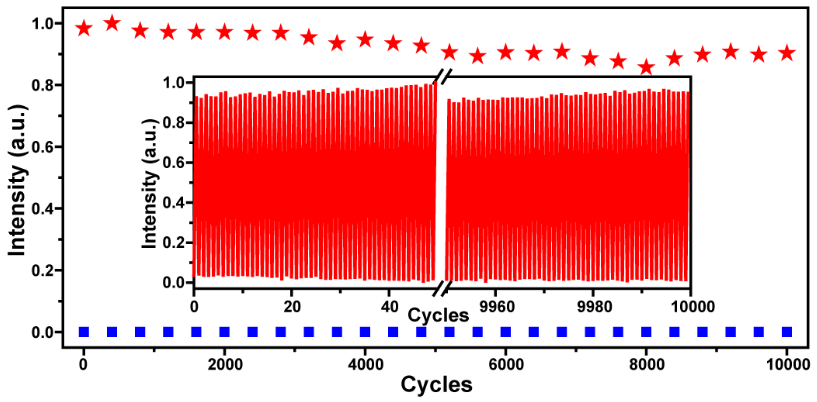

Supplementary Figure 6. The fluorescence stability (585 nm) of the Urea-N/Rh-M liquid device. Inset: The intensity of 1-50 cycles and 9950-10000 cycles of liquid device at 585 nm.

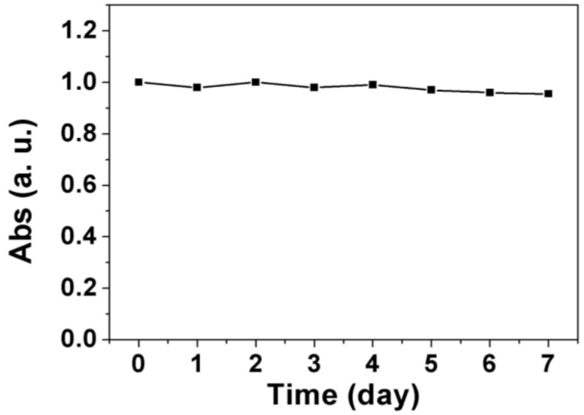

Supplementary Figure 7. The absorption change of working electrode solution separated from counter electrode solution at 560 nm with an ion-conducting membrane after being treated at 1.5 V for 20 s.

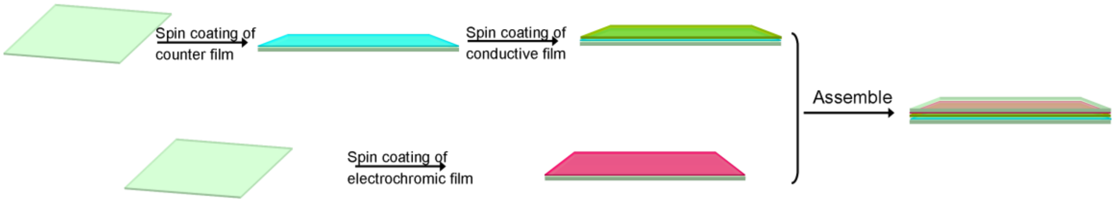

Supplementary Figure 8. The outline of the preparation of solid electrochromic device.

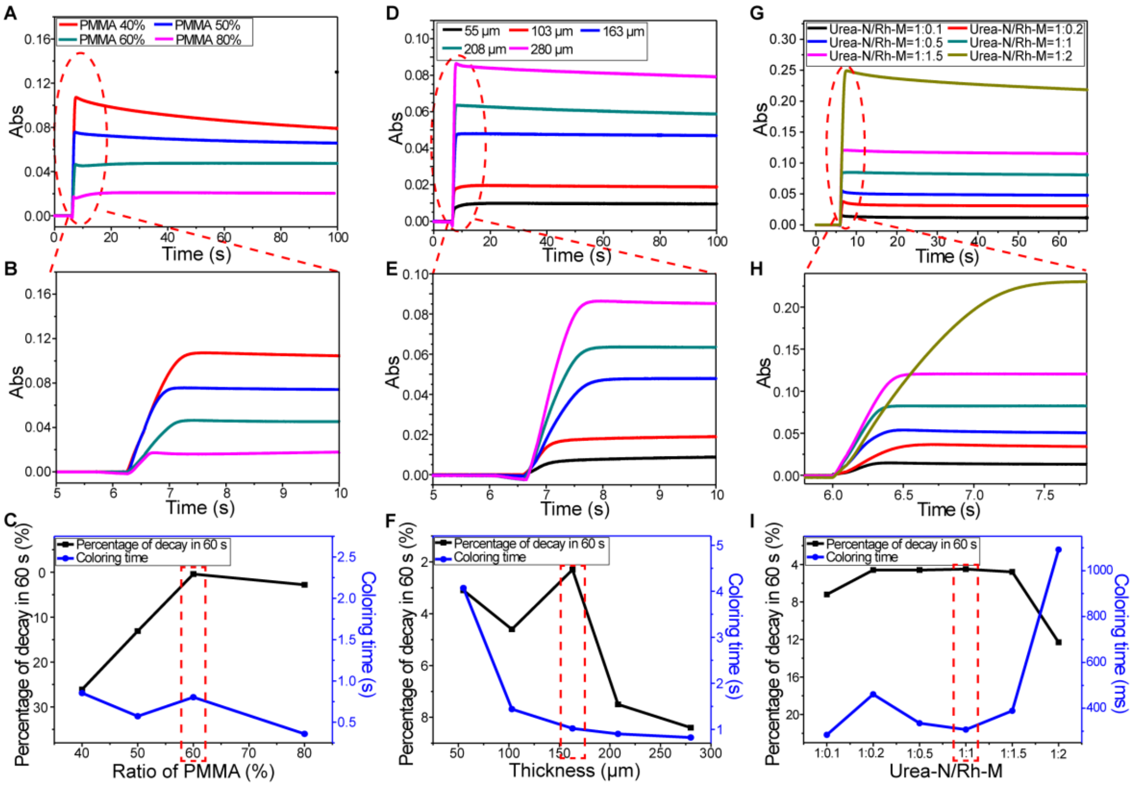

Supplementary Figure 9. The optimum conditions of the solid devices. (A) The absorption at 560 nm of solid devices with different ratio of PMMA in the electrochromic layer, counter layer, and conductive layer during the bistable process. (B) The absorption at 560 nm of solid devices during the coloring process in (A). (C) The percentage of decay in 60 s (black) and coloring time (blue) of the solid devices with different ratio of PMMA in the electrochromic layer, counter layer, and conductive layer. (D) The absorption at 560 nm of solid devices with different thickness of conductive layer during the bistable process. (E) The absorption at 560 nm of solid devices with different thickness of conductive layer during the coloring process in (D). (F) The percentage of decay in 60 s (black) and coloring time (blue) of the solid devices with different thickness of conductive layer. (G) The absorption at 560 nm of solid devices with different ratio of Urea-N/Rh-M in the electrochromic layer during the bistable process. (H) The absorption at 560 nm of solid devices with different ratio of Urea-N/Rh-M in the electrochromic layer during the coloring process in (G). (I) The percentage of decay in 60 s (black) and coloring time (blue) of the solid devices with different ratio of Urea-N/Rh-M in the electrochromic layer.

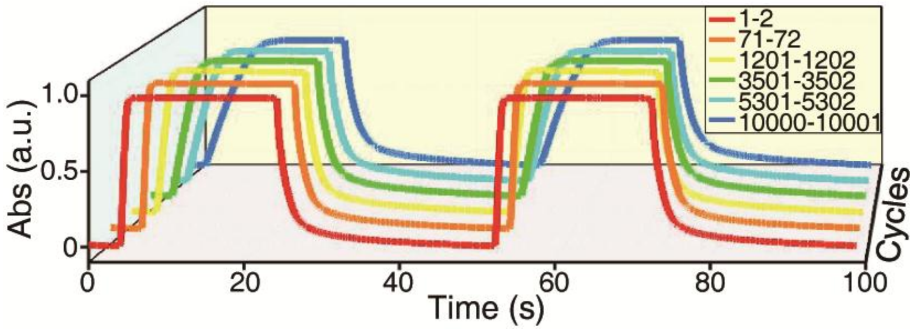

Supplementary Figure 10. The absorption of solid device at 560 nm under +1.5 V 200 ms, power off 20 s, -0.65 V 180 ms, power off 26.5 s for 10,000 cycles.

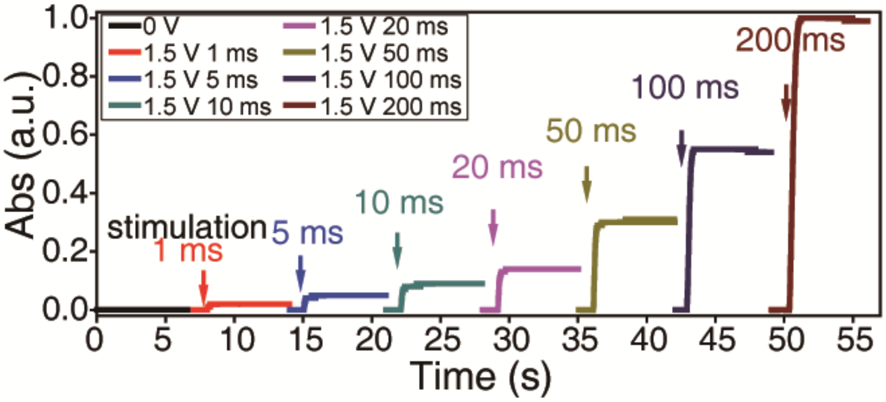

Supplementary Figure 11. The absorption at 560 nm of the solid device under 1.5 V with different stimulation time.

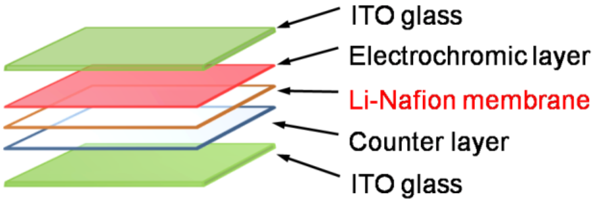

Supplementary Figure 12. The structure of Li-Nafion solid devices.

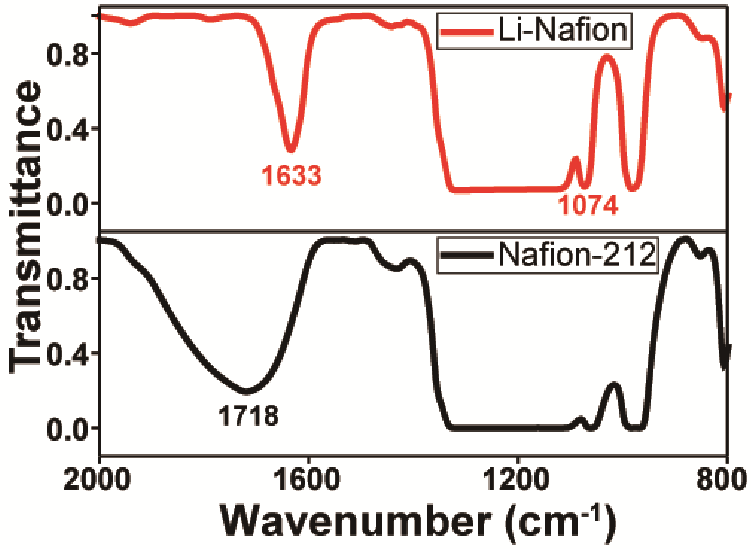

Supplementary Figure 13. The IR spectra of Nafion-212 and Li-Nafion membrane.

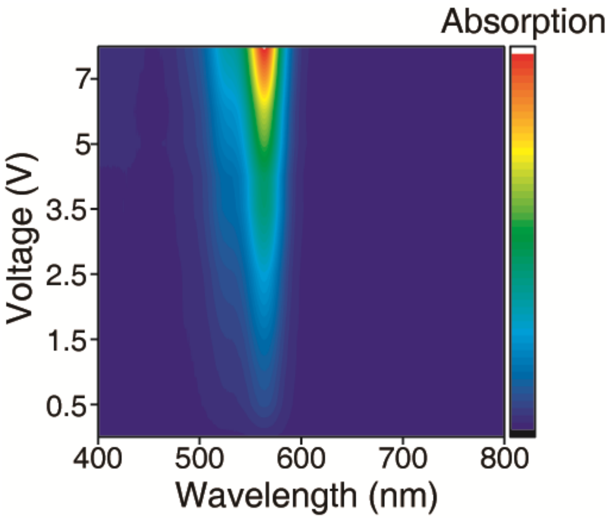

Supplementary Figure 14. The absorption spectra of the solid device with Li-Nafion as conductive layer under different voltages for 1 ms.

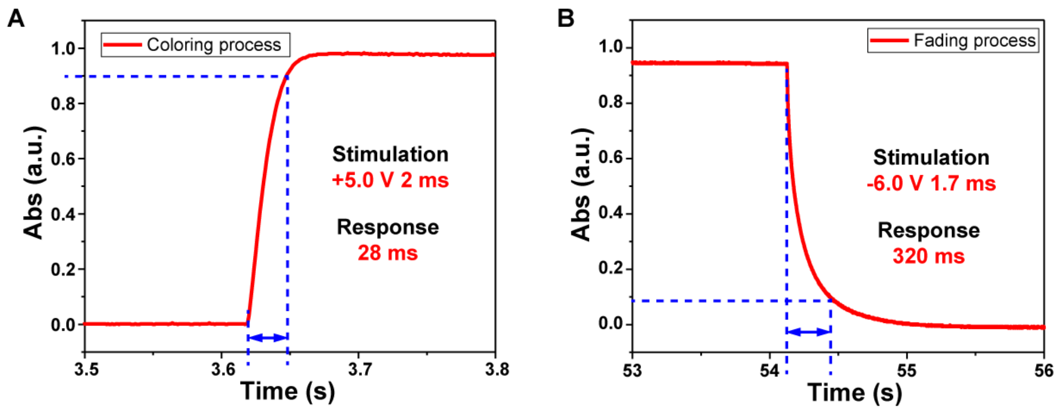

Supplementary Figure 15. The performance of coloring and fading time of Li-Nafion solid devices. (A) Coloring process of the Li-Nafion device; (B) Fading process of the Li-Nafion process.

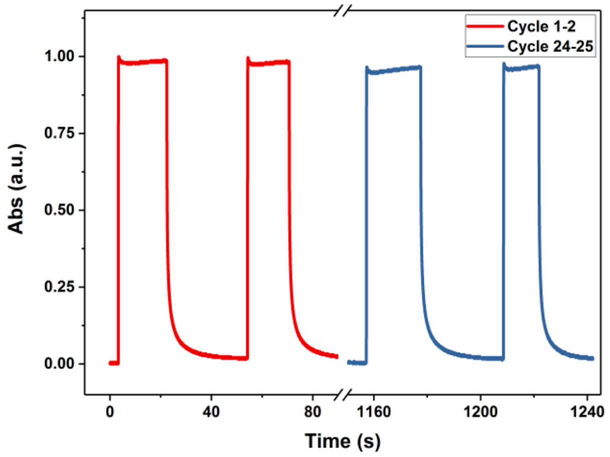

Supplementary Figure 16. The absorption spectrum of solid device with Li-Nafion conductive layer at 560 nm under +5.0 V 2 ms, wait, -6.0 V 1.7 ms (red: the 1st and 2nd cycles; blue: the 24th and 25th cycles).

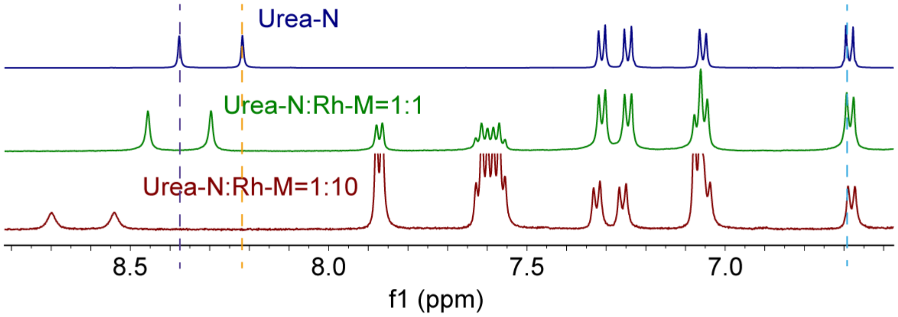

Supplementary Figure 17. ^{1}H NMR spectra (500 MHz, DMSO-d_6) of 1 eq Urea-N upon addition of Rh-M (0 eq, 1 eq and 10 eq).

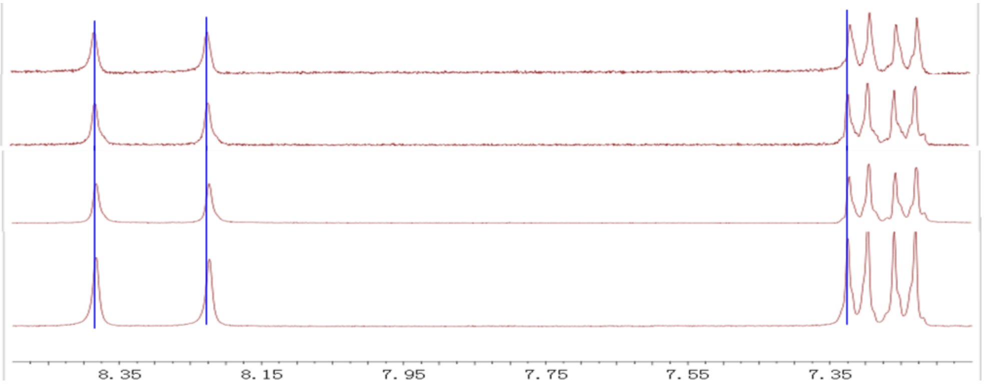

Supplementary Figure 18. **^{1}H NMR spectra (300 MHz, DMSO-*d*$_6$, 298 K) of 1 mM Urea-N of different concentration (1 mM, 2 mM, 5mM, and 10 mM).**

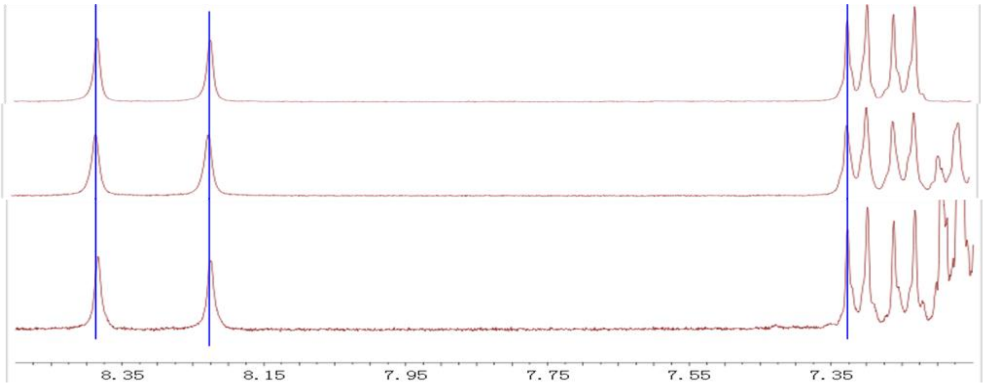

Supplementary Figure 19. **^{1}H NMR spectra (300 MHz, DMSO-*d*$_6$, 298 K) of 1 mM Urea-N upon addition of *N*,*N*-dimethylaniline (0 mM, 1 mM and 10 mM).**

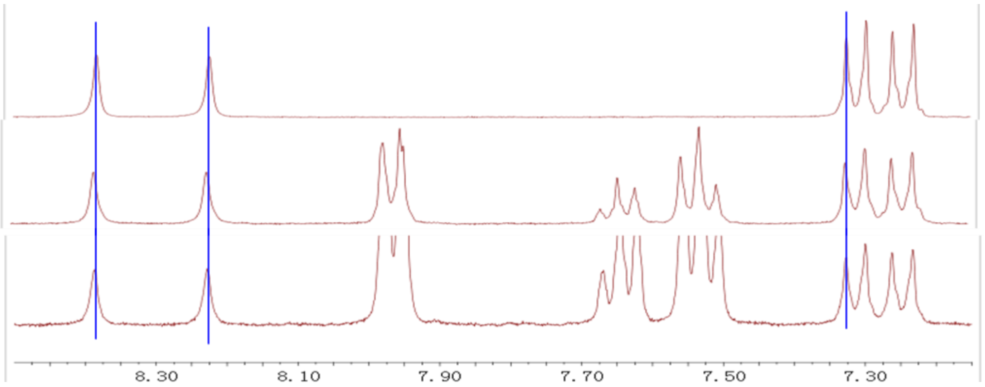

Supplementary Figure 20. ^{1}**H NMR spectra (300 MHz, DMSO-*d*$_6$, 298 K) of 1 mM Urea-N upon addition of acetophenone (0 mM, 1 mM and 10 mM).**

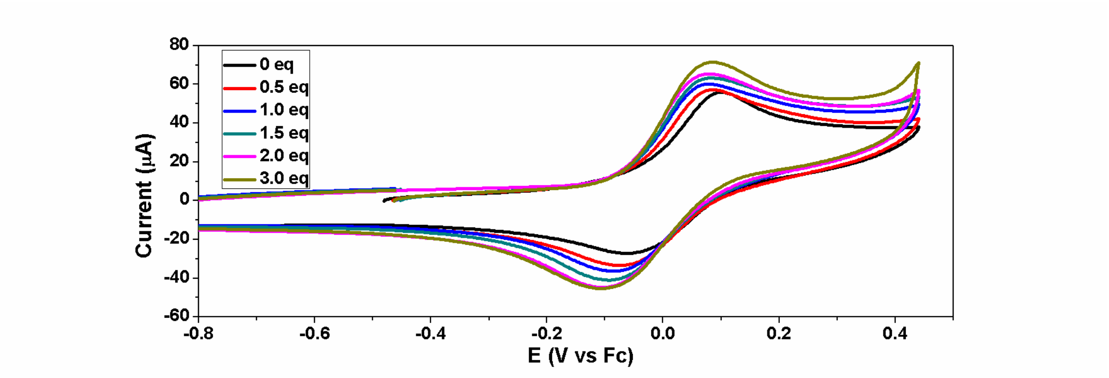

Supplementary Figure 21. Cyclic voltammogram of 1 mM Urea-N upon addition of Rh-M (0 mM, 0.5 mM and 1 mM, 1.5 mM, 2 mM, and 3 mM).

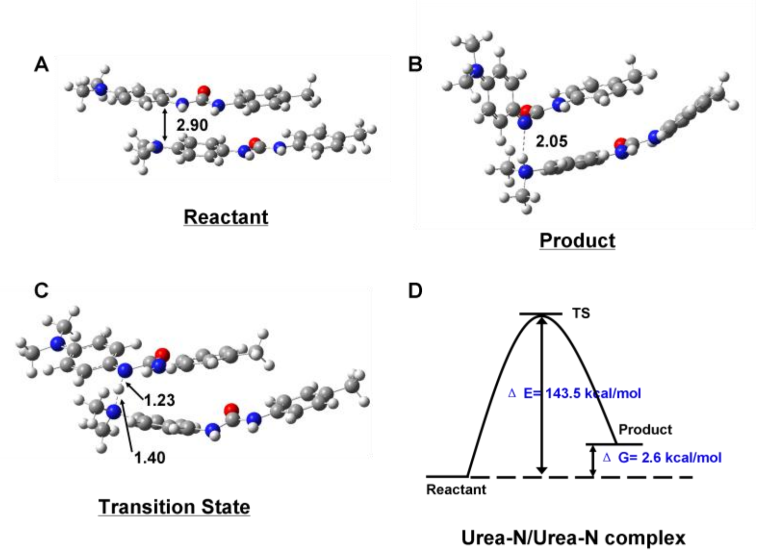

Supplementary Figure 22. The proton transfer reaction of hydrogen bond complex. Optimized structures of reactant (A), product (B) and transition state (C) of proton transfer reaction of hydrogen bond complex of **Urea-N^{2+}/Urea-N**. Potential energy diagram of the proton transfer reaction of hydrogen bond complex of **Urea-N^{2+}/Urea-N** (D).

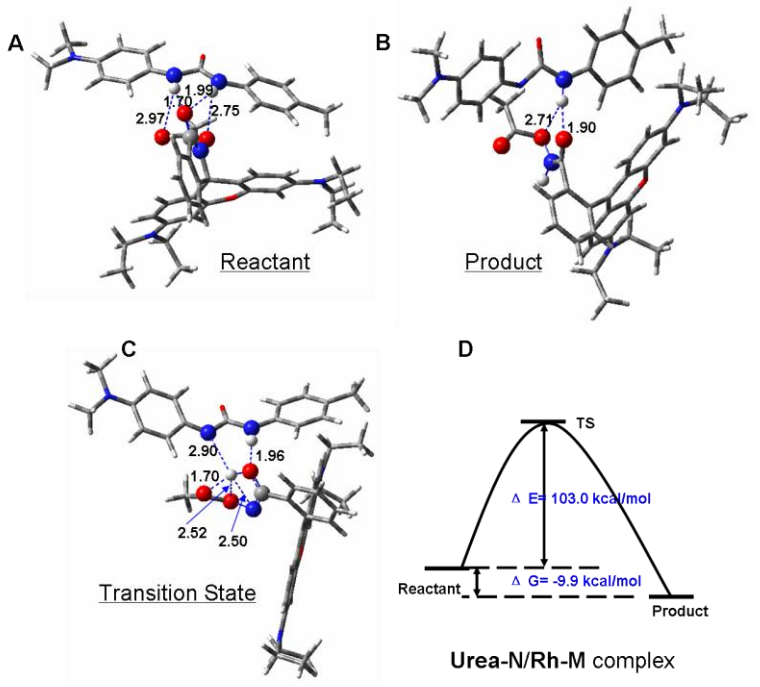

Supplementary Figure 23. The proton transfer reaction of hydrogen bond complex. Optimized structures of reactant (A), product (B) and transition state (C) of proton transfer reaction of hydrogen bond complex of **Urea-N^{2+}/Rh-M**. Potential energy diagram of the proton transfer reaction of hydrogen bond complex of **Urea-N^{2+}/Rh-M** (D).

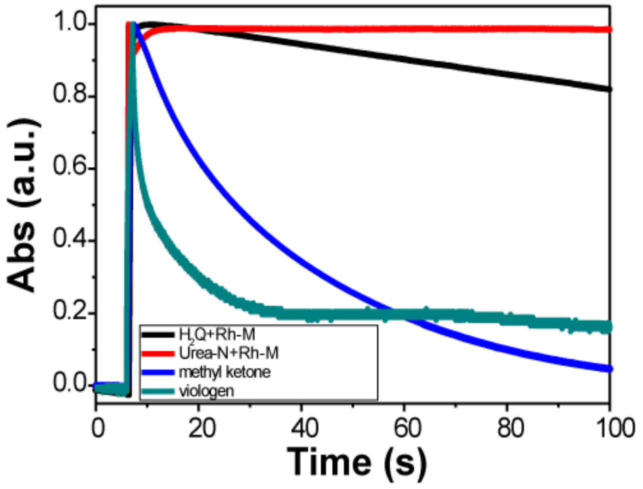

Supplementary Figure 24. The absorption intensity at 560 nm of H_2Q/Rh-M (black, 1.5 V 100 ms), Urea-N/Rh-M (red, 1.5 V 100 ms), methyl ketone (blue, -2.5 V 1 s) and viologen (green, -2.5 V 1 s) solid devices.

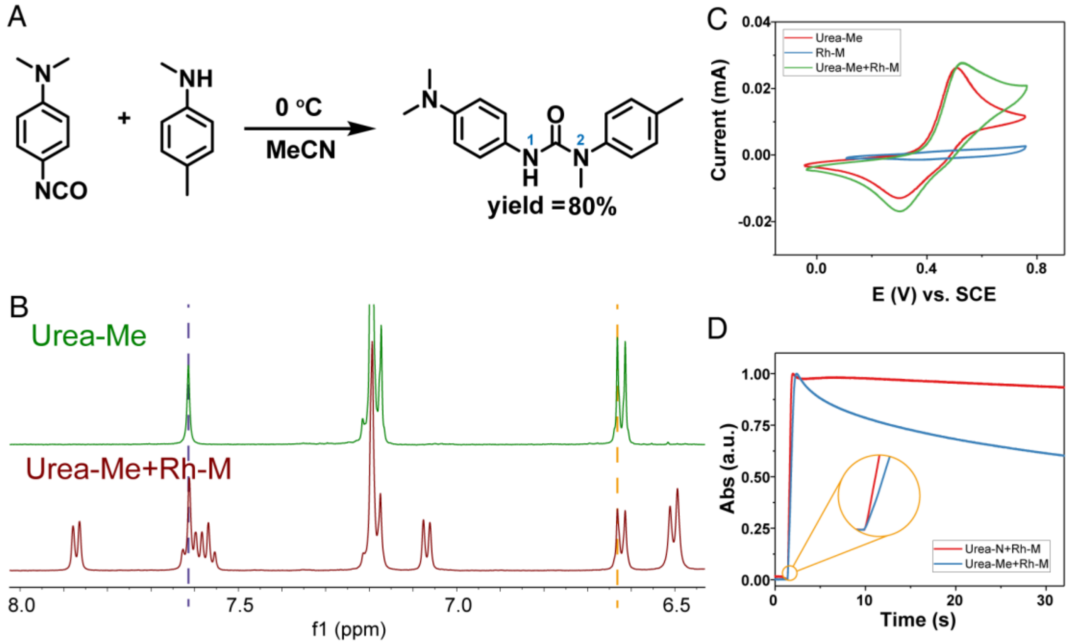

Supplementary Figure 25. The synthetic route and properties of Urea-Me. (A) The synthetic route of Urea-Me. (B) The ^{1}H NMR spectra of Urea-Me (green) and Urea-Me:Rh-M=1:1 (red). (C) The CV of Urea-Me (1.0×10^{-3} M, red), Rh-M (1.0×10^{-3} M, blue), and mixture of Urea-Me and Rh-M (green, Rh-M 1.0×10^{-3} mol Ł$^{-1}$, Urea-Me 1.0×10^{-3} mol Ł$^{-1}$). (D) The absorption spectra at 560 nm of Urea-N/Rh-M (red, 1.5 V 200 ms), Urea-Me/Rh-M (blue, 1.5 V 200 ms) solid devices.

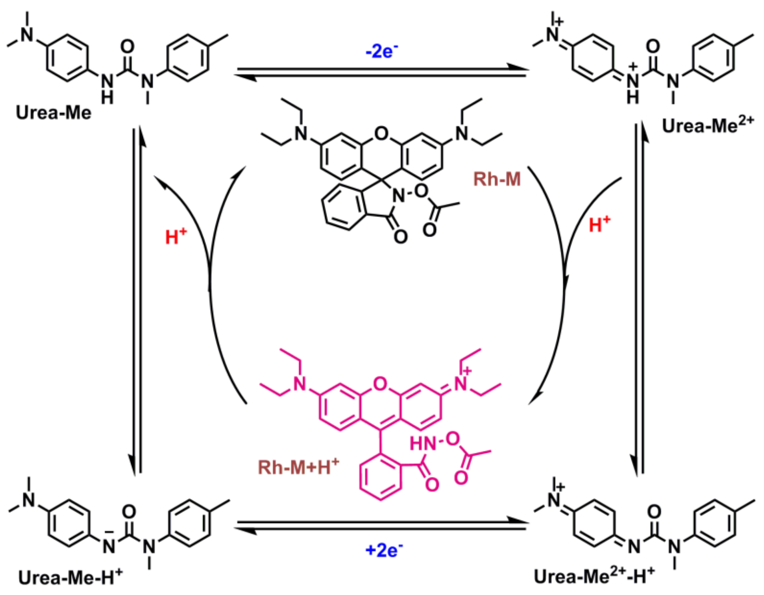

Supplementary Figure 26. The electrochromic mechanism of Urea-Me and Rh-M.

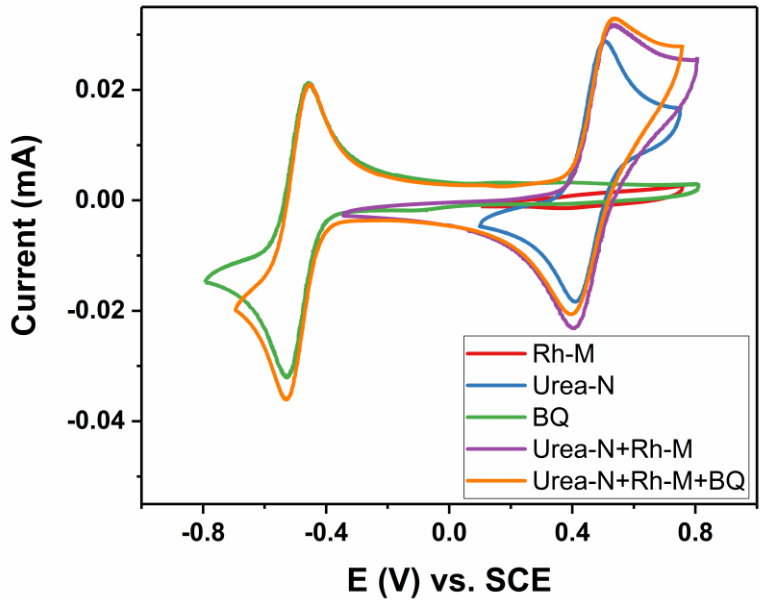

Supplementary Figure 27. Cyclic voltammogram of Rh-M (1.0×10^{-3} mol L^{-1}, red), Urea-N (1.0×10^{-3} mol L^{-1}, blue), BQ (1.0×10^{-3} mol L^{-1}, green), mixture of Rh-M and Urea-N (purple, Rh-M 1.0×10^{-3} mol L^{-1}, Urea-N 1.0×10^{-3} mol L^{-1}), and mixture of Rh-M, Urea-N and BQ (orange, Urea-N 1.0×10^{-3} mol L^{-1}, Rh-M 1.0×10^{-3} mol L^{-1}, BQ 1.0×10^{-3} mol L^{-1}).

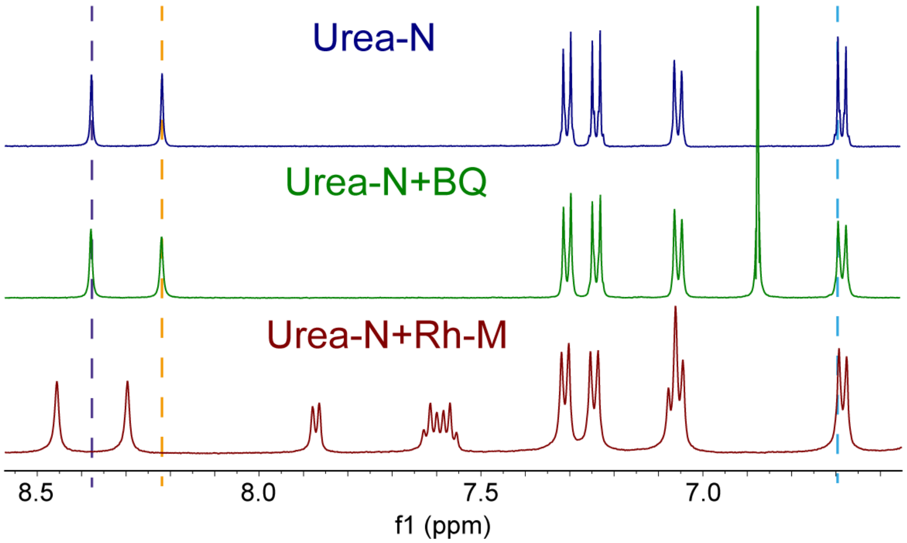

Supplementary Figure 28. The ^{1}H NMR spectra of Urea-N, Urea-N/Rh-M (1:1), and Urea-N/BQ (1:1)

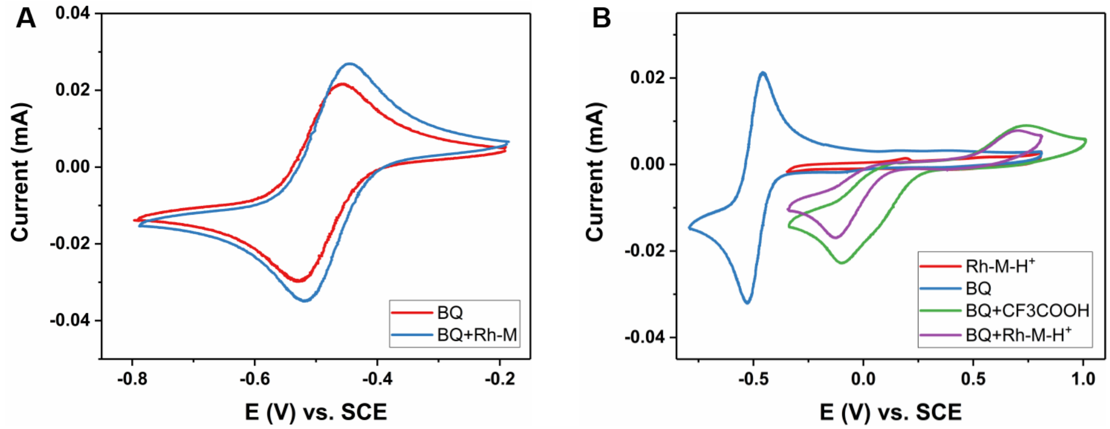

Supplementary Figure 29. The CV diagrams of (A) BQ + Rh-M (blue, BQ: 1.0×10^{-3} M, Rh-M: 1.0×10^{-3} M) and BQ (1.0×10^{-3} M, red). (B) BQ (1.0×10^{-3} M, blue), BQ+CF_3COOH (green, BQ: 1.0×10^{-3} M, CF_3COOH: 1.0×10^{-3} M), BQ+Rh-M-H^+ (purple, BQ: 1.0×10^{-3} M, Rh-M (1.0×10^{-3} M) treated with 1 eq CF_3COOH and the residue of acid was removed), and Rh-M-H^+ (red, Rh-M (1.0×10^{-3} M) treated with 1 eq CF_3COOH and the residue of acid was removed)

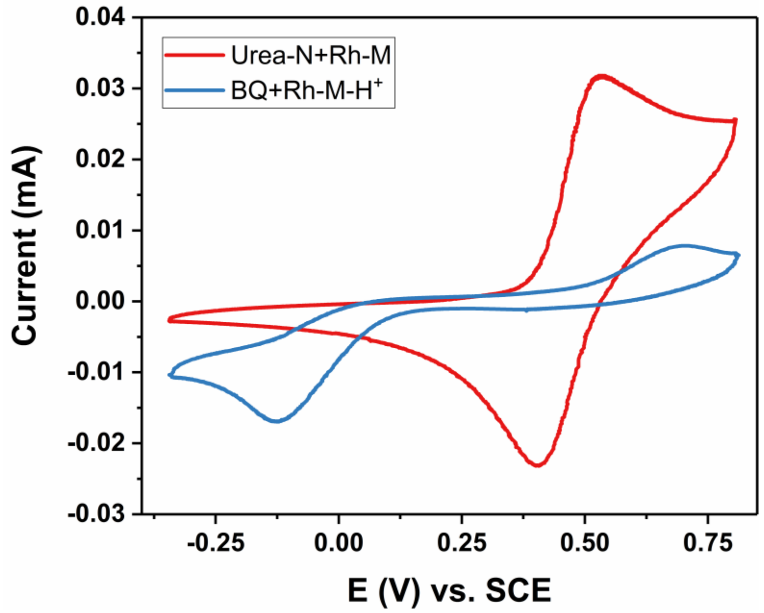

Supplementary Figure 30. The CV diagram of Urea-N + Rh-M (red, Urea-N: 1.0×10^{-3} M, Rh-M: 1.0×10^{-3} M) and BQ + Rh-M-H$^+$ (blue, BQ: 1.0×10^{-3} M, Rh-M-H$^+$: 1.0×10^{-3} M).

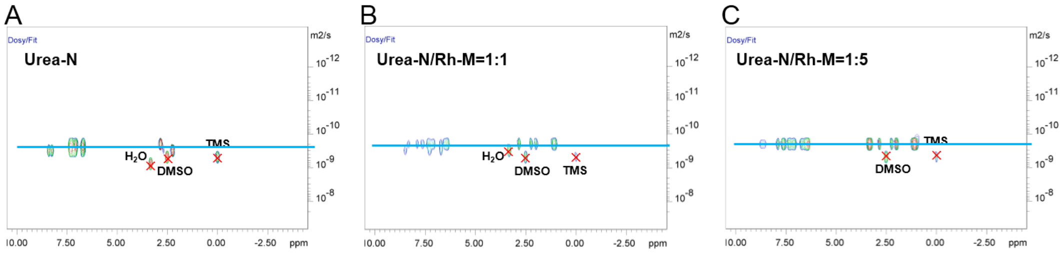

Supplementary Figure 31. DOSY spectra (500 MHz, DMSO-*d*$_6$) of (A) Urea-N, (B) Urea-N:Rh-M=1:1, and (C) Urea-N:Rh-M=1:5.

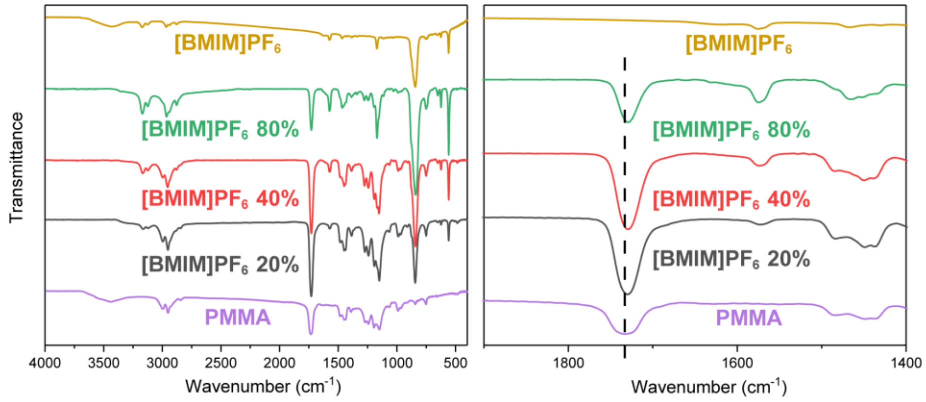

Supplementary Figure 32. The FT-IR spectra of the membrane with different ratio of PMMA/[BMIM]PF_6

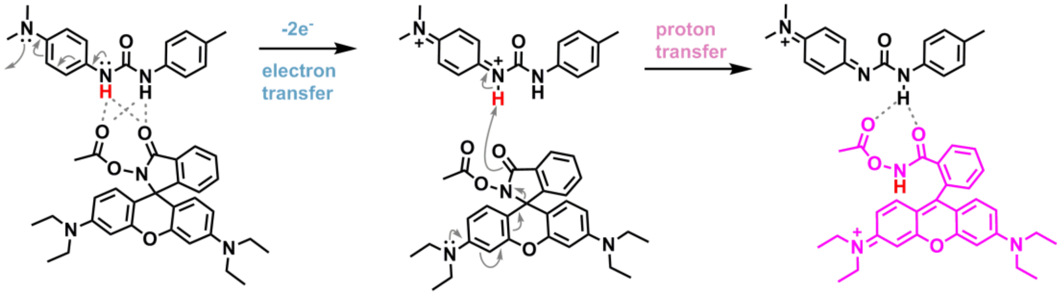

Supplementary Figure 33. The electron transfer and proton transfer process of Urea-N/Rh-M during the ring-open process.

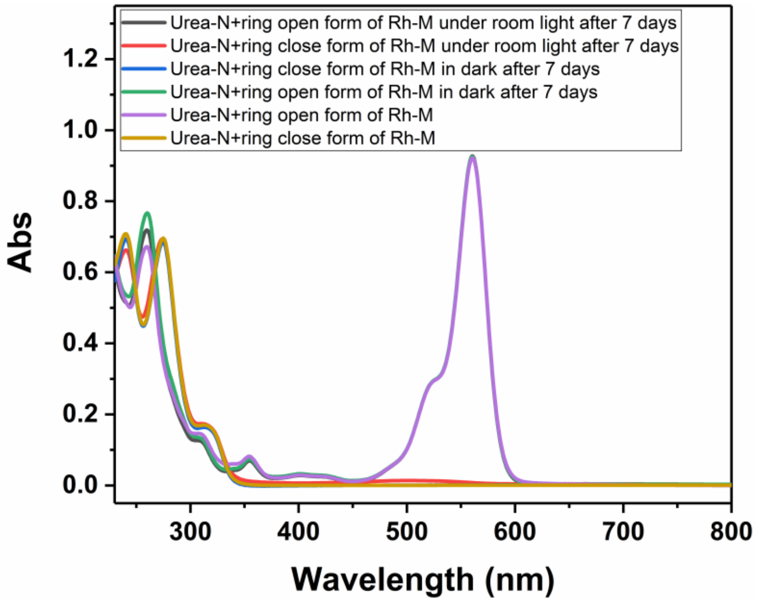

Supplementary Figure 34. The absorption spectra of the Urea-N+ring open/close form of Rh-M and the spectra of the Urea-N+ring open/close form of Rh-M in dark/under room light after 7 days.

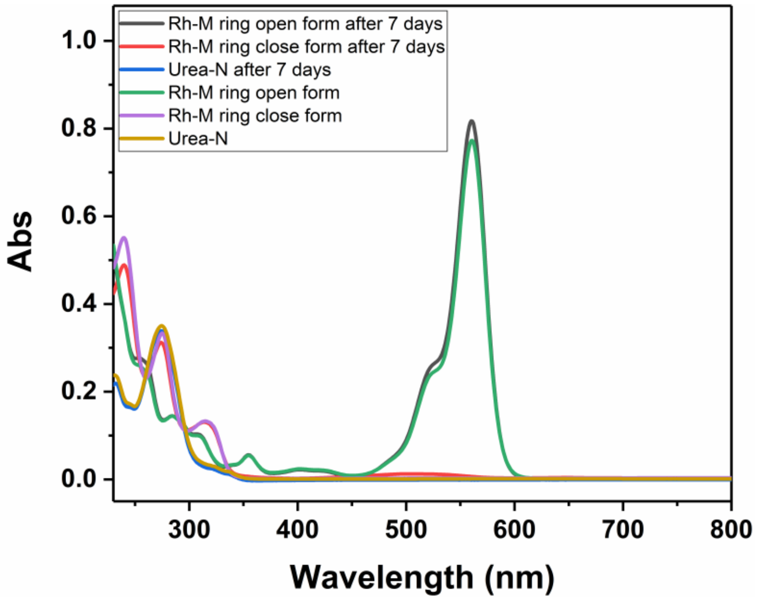

Supplementary Figure 35. The absorption spectra of ring open form of Rh-M, ring close form of Rh-M, Urea-N and their spectra after 7 days.

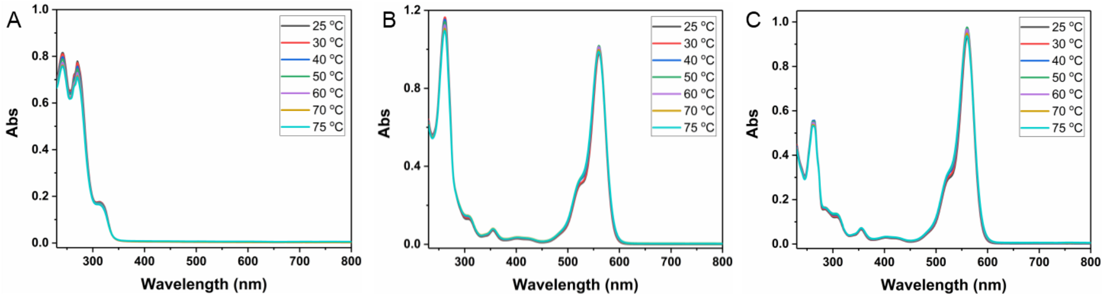

Supplementary Figure 36. The absorption spectra of (A) Rh-M + Urea-N (Urea-N:1.0×10^{-5} M; Rh-M:1.0×10^{-5} M); (B) Rh-M + Urea-N with 1 eq CF_3COOH (Urea-N:1.0×10^{-5} M; Rh-M:1.0×10^{-5} M; CF_3COOH: 1.0×10^{-5} M); (C) Rh-M with 1 eq CF_3COOH (Rh-M:1.0×10^{-5} M; CF_3COOH: 1.0×10^{-5} M) under different temperature.

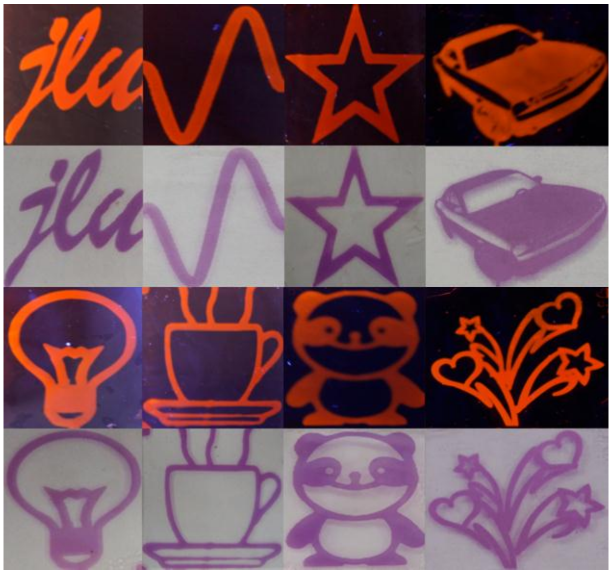

Supplementary Figure 37. Photographs of the electrochromic and electrofluorochromic patterns on the solid device.

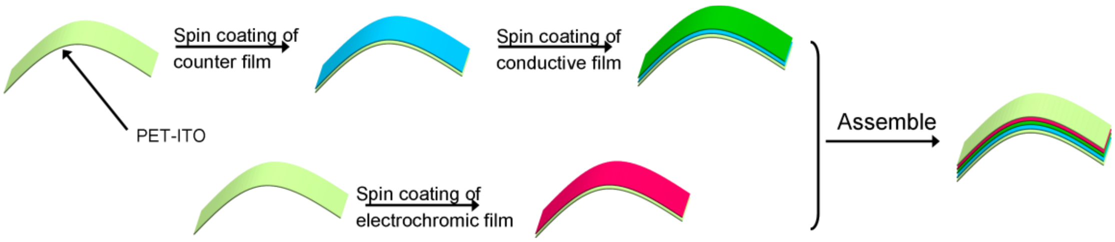

Supplementary Figure 38. The outline of the preparation of solid flexible electrochromic device.

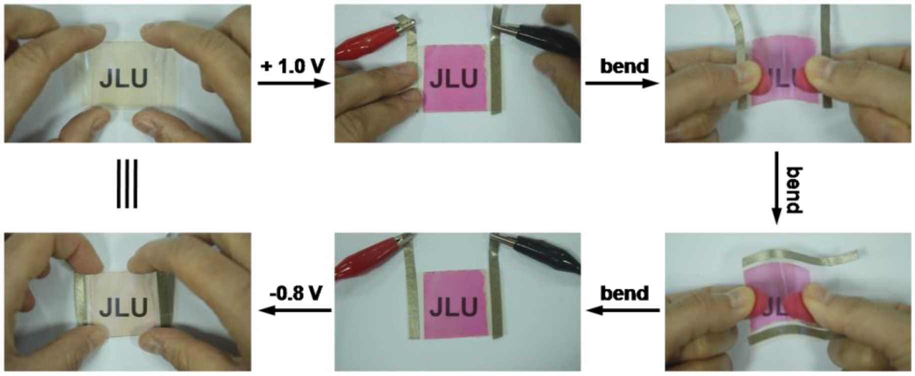

Supplementary Figure 39. Photographs of the flexible solid electrochromic device.

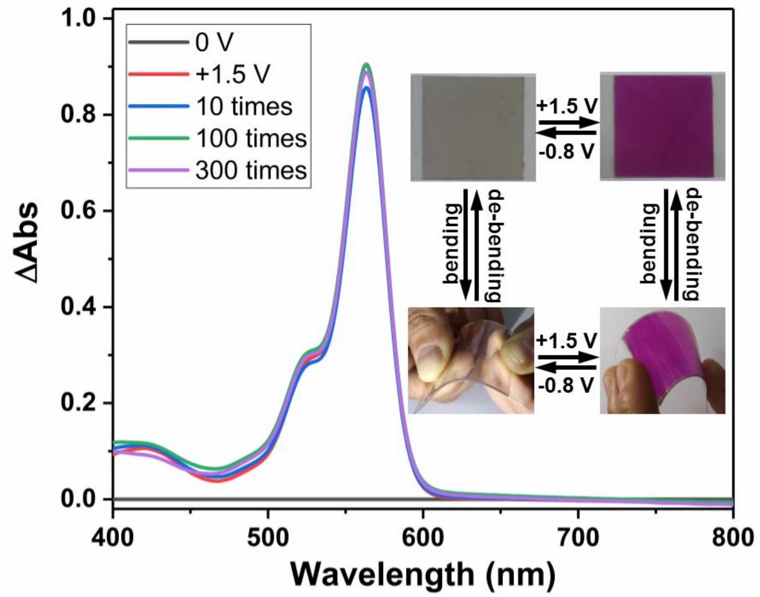

Supplementary Figure 40. The absorption spectra of flexible solid devices on different bending times. Inset: the photos of flexible solid device.

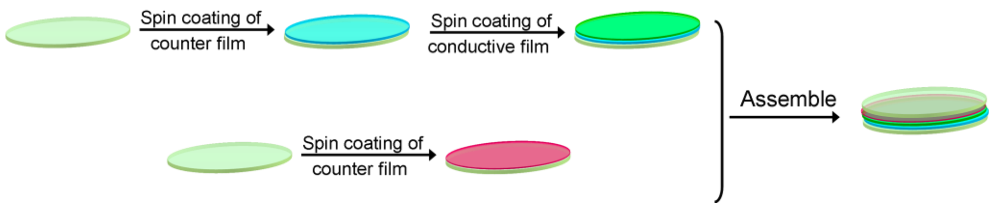

Supplementary Figure 41. The outline of the preparation of the smart glasses.

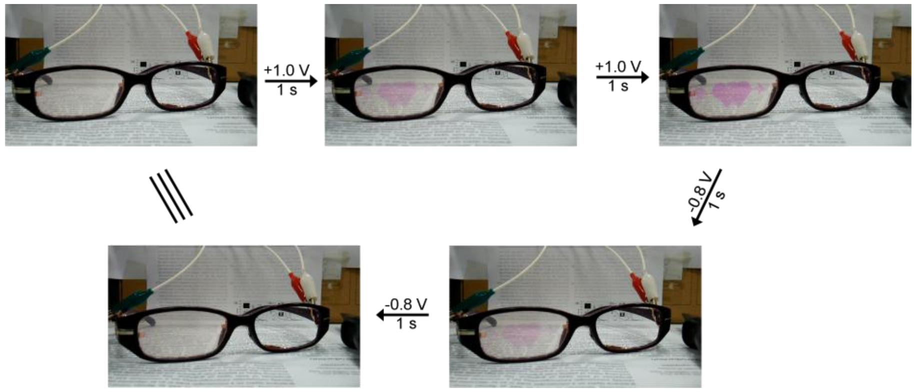

Supplementary Figure 42. Photographs of a prototype as smart glasses.

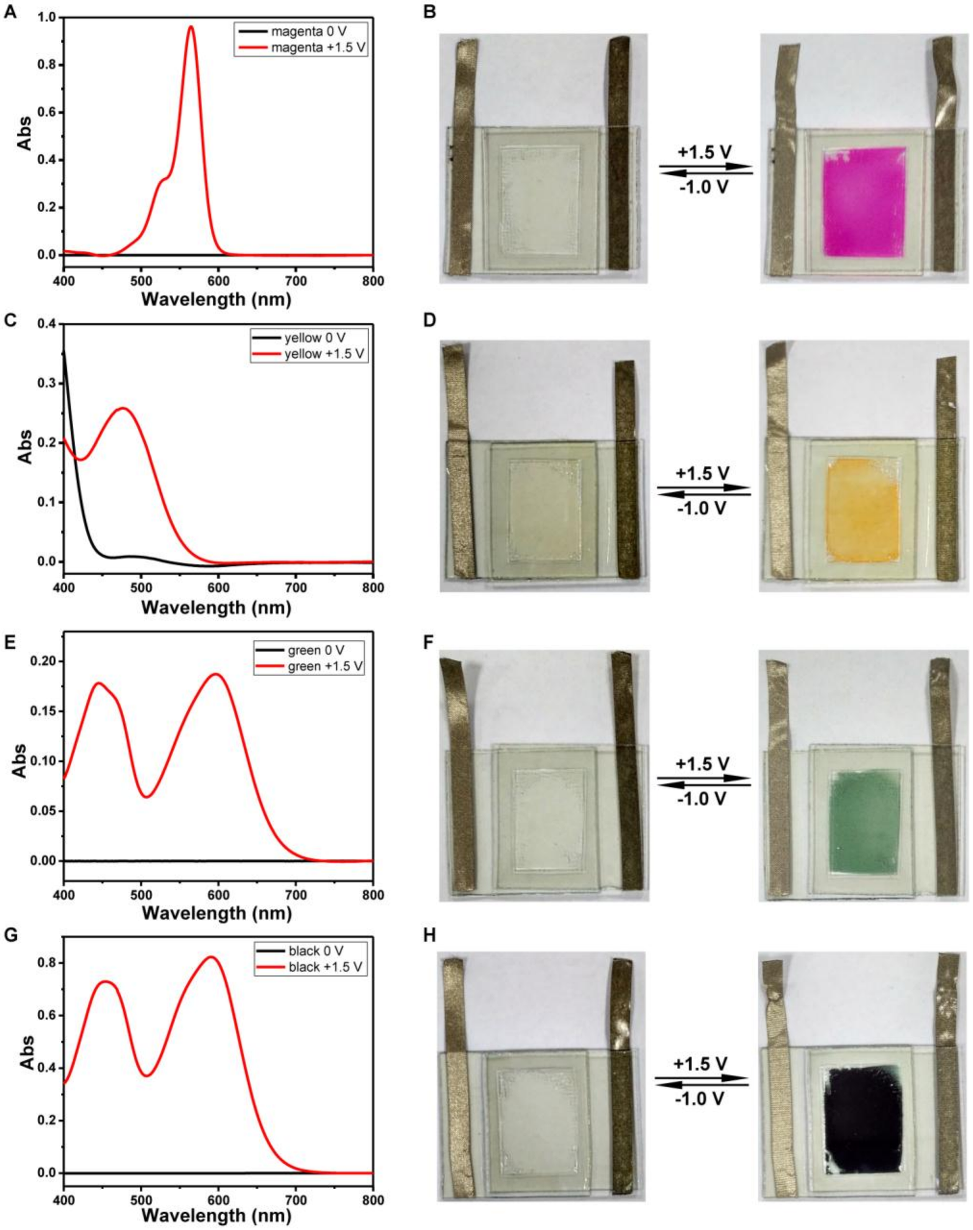

Supplementary Figure 43. UV-vis spectra and photographs of multicolor bistable electrochromic solid devices. The UV-vis spectra (A) and photos (B) of magenta bistable device under 0 V and +1.5 V. The UV-vis spectra (C) and photos (D) of yellow bistable device under 0 V and 1.5 V. The UV-vis spectra (E) and photos (F) of green bistable device under 0 V and +1.5 V. The UV-vis spectra (G) and photos (H) of black bistable device under 0 V and 1.5 V.

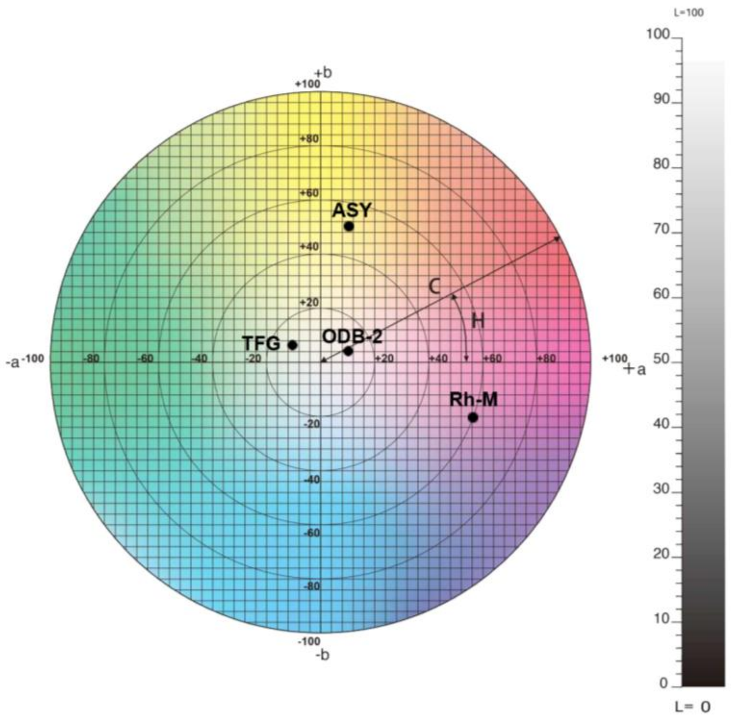

Supplementary Figure 44. The chromaticity coordinates of the devices with different pH sensitive dyes under the bias voltage of +1.5 V in the chromaticity diagram.

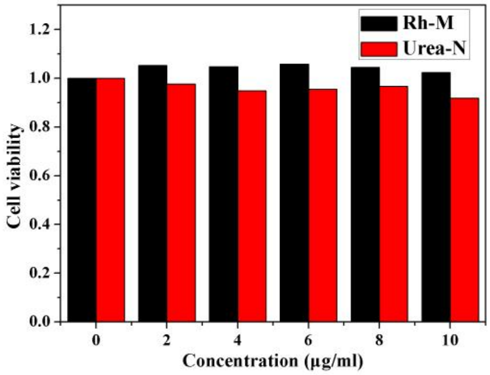

Supplementary Figure 45. Result of cell cytotoxicity test of Rh-M and Urea-N.

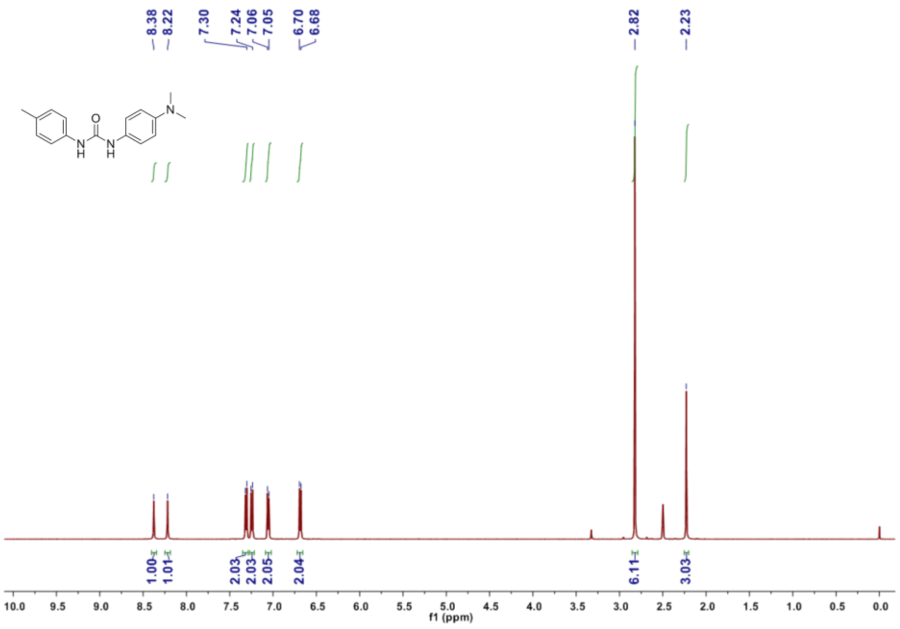

Supplementary Figure 46. **^{1}H NMR (500 MHz, DMSO-*d_6*) spectra of Urea-N**

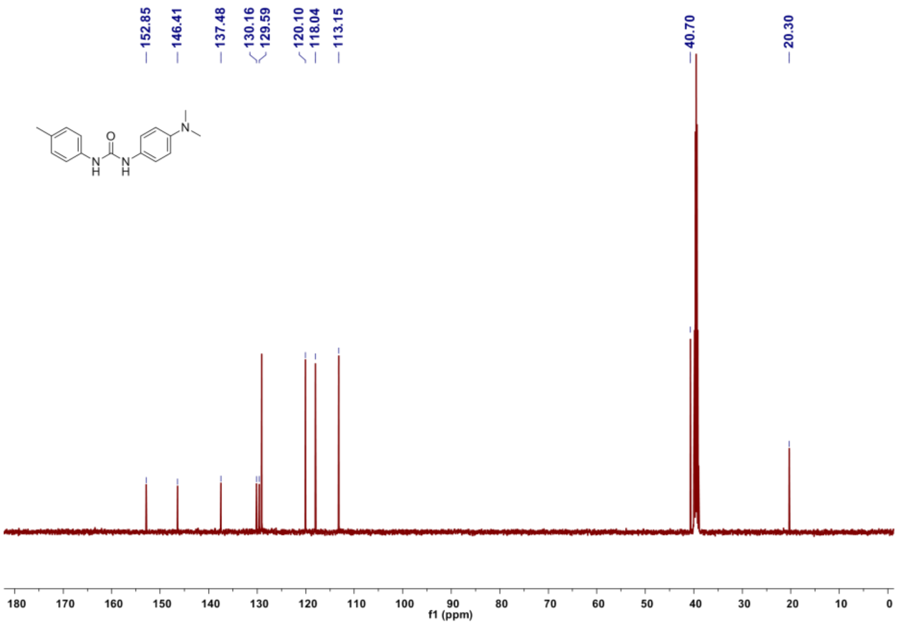

Supplementary Figure 47. **^{13}C NMR (126 MHz, DMSO-*d_6*) spectra of Urea-N**

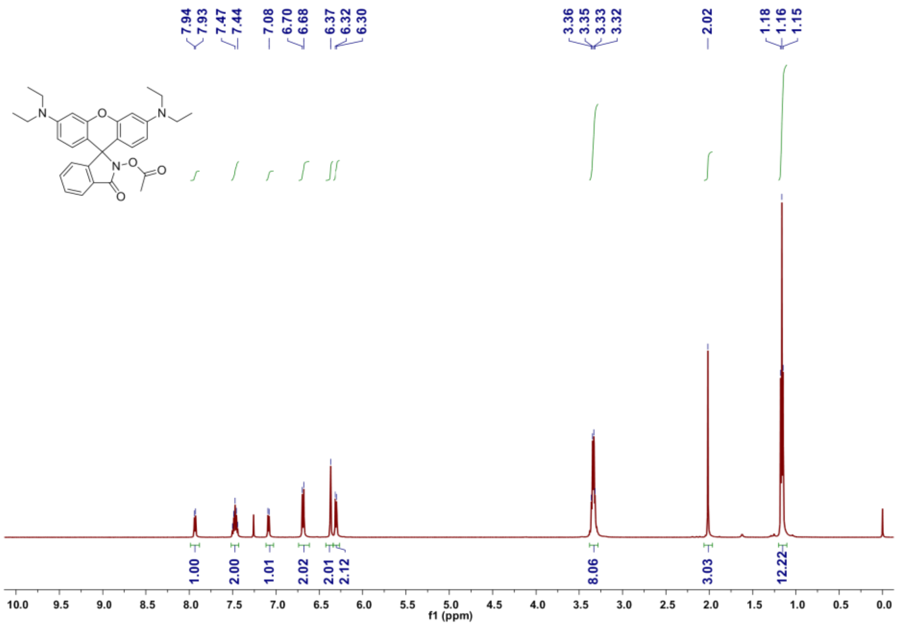

Supplementary Figure 48. ^{1}H NMR (500 MHz, $CDCl_3$) spectra of Rh-M

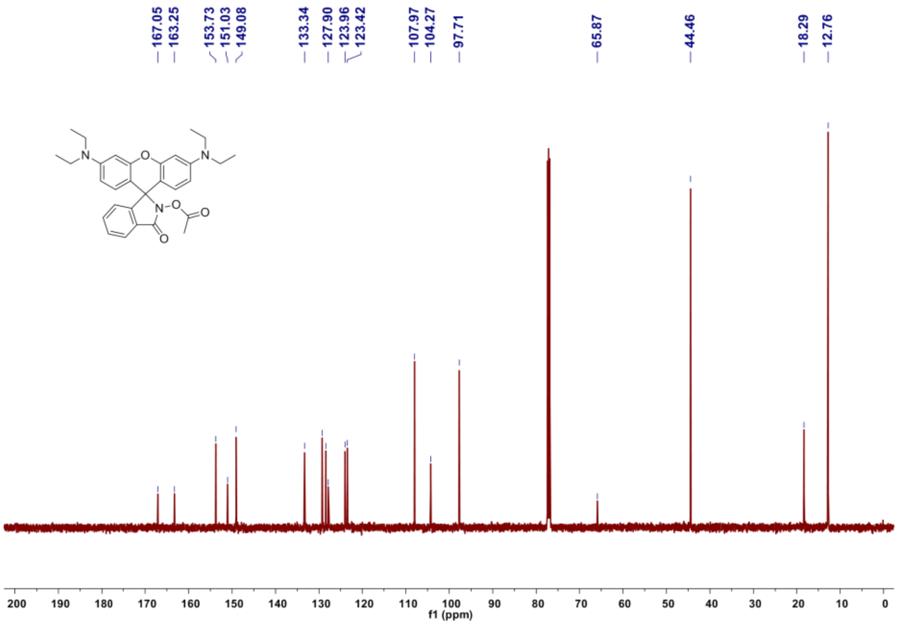

Supplementary Figure 49. ^{13}C NMR (126 MHz, $CDCl_3$) spectra of Rh-M

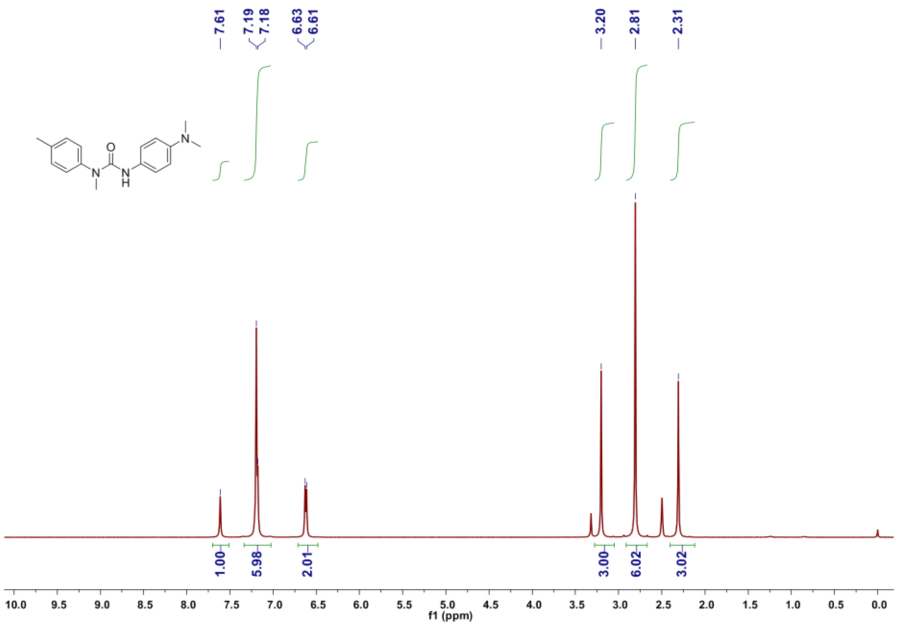

Supplementary Figure 50. **^{1}H NMR (500 MHz, DMSO-d_6) spectra of Urea-Me**

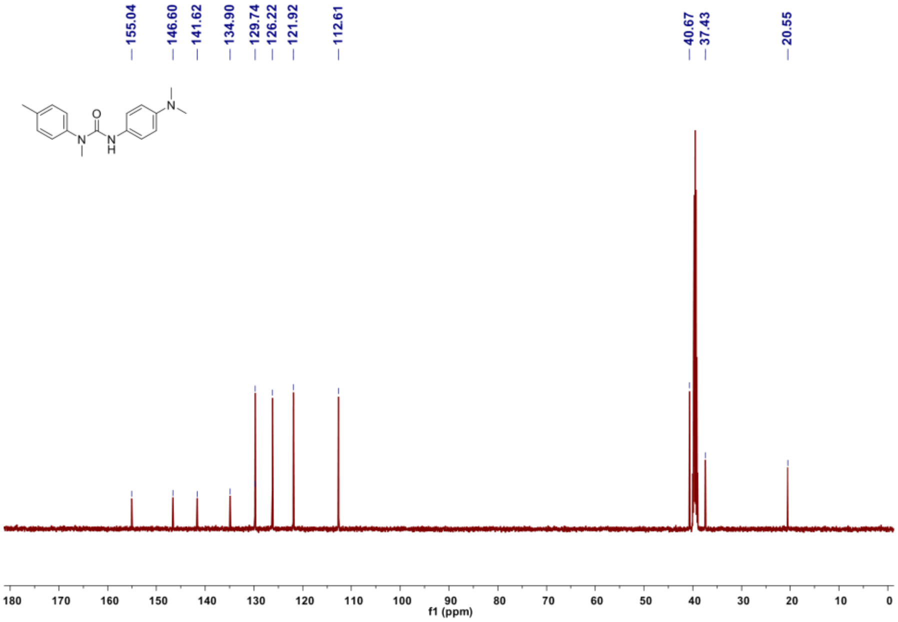

Supplementary Figure 51. **^{13}C NMR (126 MHz, DMSO-d_6) spectra of Urea-Me**

Supplementary Table 1. Summary of crystal data and intensity collection parameters for Rh-M

Compound	Rh-M
**Formula**	**$C_{30}H_{33}N_3O_4$**
**Formula mass**	**499.59**
**Space group**	**triclinic, P-1**
**a/Å**	**11.453(2)**
**b/Å**	**11.637(2)**
**c/Å**	**12.216(2)**
**α/º**	**80.16(3)**
**β/º**	**63.01(3)**
**γ/º**	**65.49(3)**
**V/ $Å^3$**	**1319.9(5)**
**Z/mg.m^{-3}**	**2, 1.257**
**F_{000}**	**532**
**Theta range/º**	**3.39 to 27.48**
**No. of collected reflns**	**13021 / 5985**
**No. of unique reflns.(R_{int})**	**0.0472**
**Data/restrains/parameters**	**5985 / 0 / 339**
**R_1,wR_2[obs I>2σ(I)]**	**R1 = 0.0586, wR2 = 0.1587**
**R_1,wR_2 (all data)**	**R1 = 0.1024, wR2 = 0.1895**
**Residual peak/hole e. $Å^{-3}$**	**0.299 and -0.172**
**CCDC number**	**1897574**

Supplementary Table 2. The diffusion coefficient of Urea-N with different equivalents of Rh-M tested by DOSY

	**Urea-N**	**Urea-N/Rh-M 1:1**	**Urea-N/Rh-M 1:5**
**D / m^2 s^{-1}**	2.45×10^{-10}	2.48×10^{-10}	2.40×10^{-10}

Supplementary Table 3. The conductivity and photographs of PMMA film with different percentage of ionic liquid

Percentage of ionic liquid	60%	40%	20%
Conductivity (S cm^{-1})	1.7×10^{-5}	3.5×10^{-6}	3.1×10^{-7}
Photographs	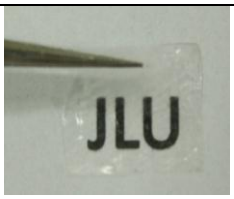	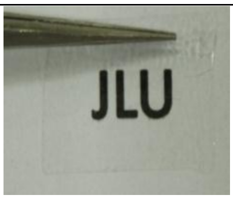	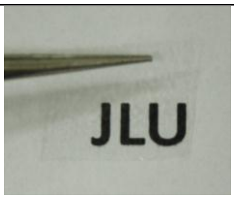

Supplementary Table 4. The CIE L*a*b* value of different solid devices with different pH sensitive dyes under the bias voltage of +1.5 V

Acid sensitive molecules	Rh-M	ASY	TFG	ODB-2
color	**Magenta**	**Yellow**	**Green**	**Black**
L*	**36.88**	**57.35**	**48.81**	**8.77**
a*	**56.69**	**10.31**	**-10.10**	**10.42**
b*	**-20.71**	**51.21**	**6.98**	**4.44**

Supplementary Note 1. Light and thermal stability of Urea-N and Rh-M

In order to investigate the stability of the electrochromic materials under light irradiation, the UV-vis spectra of the mixture of Urea-N/Rh-M under room light and in dark were measured respectively. According to Supplementary Figure 34, the Urea-N/Rh-M mixture shows good stability on both ring-open and ring-close state of Rh-M when exposed to the room light, for the absorption spectra have no obvious change during 7 days, which indicates the good stability of the EC molecules under room light.

In order to test the thermal stability of the electrochromic materials, the UV-vis absorption spectra of both ring-open state and ring-close state of Rh-M + Urea-N, and Rh-M with 1 eq CF_3COOH were measured under different temperature. The spectra in Supplementary Figure 36 showed that the absorption shows little change under different temperature from 25 ^oC to 75 ^oC, which indicates a good thermal stability of our materials at both ring-open and ring-close state, and the thermal stability provides the potential for future application to some extent.

Supplementary Note 2. Electrofluorochromic performance in liquid devices

For small organic electrochromic materials, the coloration efficiency, η, is defined by the change of injected charges per unit area (ΔQ) and the optical absorbance change (ΔA). And the η for the liquid device is about 150 $cm^2\ C^{-1}$, as shown in Supplementary Figure 5d. More importantly, electrofluorochromic switching can be repeated 10000 times with no sign of degradation by consecutive on/off switching cycles (Supplementary Figure 6), which indicates that this device is extremely durable.

Supplementary Note 3. Optimum parameters of the solid device

Here we propose to use ionic liquid (1-butyl-3-methylimidazolium hexafluorophosphate, $[BMIM]PF_6$) in PMMA for substituting both functions of electrolyte and plasticizer in our device. As a result, excellent conductivity (3.5×10^{-6} S cm^{-1}, as shown in Supplementary Figure Table 3) and transparency with good mechanical strength and compressive stress were obtained.

Different factors such as ratio of PMMA/$[BMIM]PF_6$, thickness of conductive layer and ratio of Urea-N/Rh-M were investigated to obtain optimum parameter for the bistable solid device. (Supplementary Figure 9)

In order to fabricate the devices with good bistability, fast response time and fading time, different factors such as ratio of PMMA/$[BMIM]PF_6$, thickness of conductive layer and ratio of Urea-N/Rh-M were investigated. Supplementary Figure 9a-c show the bistable and coloring properties of solid devices with different ratio of PMMA in the electrochromic layer, counter layer, and conductive layer. Within the range of ratio of PMMA between 40% to 80%, the fluidity of the three layers turns down and the bistability of the device turns better but the intensity of absorption at 560 decreases with the increase of the ratio of PMMA, as Supplementary Figure 9a shown. According to Supplementary Figure 9a and b, the percentage of decay in 60 s (Supplementary Figure 9c, black) and the coloring time (Supplementary Figure 9c, blue) can be read, the ratio of 60% and 80% exhibit good stability on colored state and short coloring time, which is suitable for bistable electrochromic device. However, the intensity is too

weak when the ratio of PMMA is 80% (pink line in Supplementary Figure 9a and b). Thus, the appropriate condition of PMMA ratio is 60%. As for Supplementary Figure 9d-f, the bistable and coloring properties of solid devices with different thickness of conductive layer are investigated. Considering both bistability and coloring time, the thickness of 163 μm shows low percentage of decay and short coloring time among the five sets of data. Therefore, the proper condition of different thickness of conductive layer is around 163 μm. According to Supplementary Figure 9g-i, with the ratio range from 1:0.1 to 1:1.5, all the devices show excellent bistability, with the increase of ratio of Urea-N/Rh-M, the intensity of absorption increases. As for the coloring time, the devices exhibit short coloring time between the ratio of 1:0.1 to 1:1.5, compared with the ratio of 1:2 (1200 ms). Considering both the two aspects, the ratio of 1:1 is chosen as the best ratio of Urea-N/Rh-M. The influences of each factor on coloring time, fading time and the percentage of decay were investigated and PMMA/[BMIM]PF_6=6:4, Urea-N/Rh-M=1:1 and thickness of conductive layer between 150 and 200 μm were considered as the optimum conditions according to the experiments above.

Supplementary Note 4. The electrochromic mechanism

The mechanism of BQ as counter material in EC layer

In order to shorten the fading time of the device when under negative voltages, BQ was added to electrochromic layer due to its electrobase properties. In order to research the interaction among the three molecules, we tested the cyclic voltammetry and ^{1}H NMR spectra of the sole molecule and mixture under different conditions.

According to Supplementary Figure 27, when applied positive voltages, an oxidation peak appeared in both the mixture of Urea-N and Rh-M (Supplementary Figure 28, purple) and mixture of Urea-N, Rh-M and BQ (Supplementary Figure 28, orange), which was similar to the oxidation peak of Urea-N (Supplementary Figure 28, blue), while the solution with sole Rh-M and BQ had no redox peak between 0 V to +0.8 V. The CV diagrams reveal that the color change of pH sensitive dye Rh-M results from the oxidation of Urea-N to release proton under positive voltages. However, according to Supplementary Figure 27 (green curve), under such 0 V – 0.8 V positive voltage range, the reduction of BQ only occurs on counter electrode, and the neutral state BQ around working electrode is likely unable to capture the proton which is released by oxidized Urea-N. Meanwhile, in Supplementary Figure 28, the ^{1}H NMR spectra also demonstrate the interaction between Urea-N and Rh-M is stronger than that between Urea-N and “BQ in neutral state”. There are Urea-N, Rh-M and BQ in the EC layer of solid bistable device. Thus, when device changes from colorless to colored state, the main reaction in the EC layer occurs between Urea-N and Rh-M rather than BQ. And although there is BQ as counter material in device, the EC layer and counter layer are separated by a conductive and isolating membrane of conductive Li-Nafion or PMMA/[BMIM]PF_6, which effectively prevent the contact and diffusion of the electroactive molecules from the other two layers to some extent, and consequently avoids the redox self-discharging reaction.

As Supplementary Figure 29 and Supplementary Figure 30 shown, when oxidized Urea-N, Rh-M-H^+ and reduced BQ coexist in the device, the reduced benzoquinone will compete with Rh-M to accept the proton, which supports the perspective of the reviewer. When colored device begins to fade under negative voltage, the main active species in the electrochromic device are

BQ, Rh-M-H^+, and oxidized Urea-N. According to Supplementary Figure 30a, the redox peak of sole BQ and mixture of BQ and Rh-M show little change, however, when mixed with Rh-M-H^+ (Rh-M treated with 1 eq CF_3COOH and then the residue of acid was removed), the reduction peak of BQ shifts 0.4 V (from -0.53 V to -0.13 V). Notably, the shape of the redox peak of BQ also changes when mixed with Rh-M-H^+ (Supplementary Figure 29b). The shift of the reduction peak of BQ and the departure of the redox peak reveals the proton transfer between the two components according literature[1],[2], which is to say BQ can capture the proton from the Rh-M+H^+. The redox peak of BQ/Rh-M+H^+ is similar to that of BQ/CF_3COOH, which provides another evidence of the proton transfer between BQ and Rh-M+H^+. The proton transfer can help accelerate the fading speed of the device to some extent. Compared with the redox peak of Urea-N/Rh-M (Supplementary Figure 30), the potential of reduction peak of BQ/Rh-M+H^+ is much lower and the oxidation peak is much higher. It can be inferred from the phenomena above that reduced BQ is able to give proton back to oxidized Urea-N to form neutral BQ and Urea-N after capturing proton from Rh-M+H^+, which makes the system totally reversible.

Comparison of different EC materials

In order to further prove the mechanism we proposed, the methyl ketone bridged molecule, viologen, H_2Q/Rh-M was applied in EC layer of the device instead of Urea-N/Rh-M. As Supplementary Figure 24 shown, the methyl ketone bridged molecule and viologen shows poor bistability in the solid device and the H_2Q/Rh-M decays about 20% during 100 s, while the Urea-N/Rh-M device exhibits superb bistability. When the mechanisms of the four systems are futher investigated, the differences among the four systems are figured out. For the viologen molecule, the redox of the viologen directly leads to the absorption and color change of the device, and there is no proton transfer process in the system. As for the methyl ketone molecule, there are both electron transfer and proton transfer, however, there is no hydrogen bonding in the system, which is proved to help stabilizing the electrochromic molecules. For the two electroacid systems, although they both have the electron transfer and proton transfer during the process, and there are hydrogen bonding in both systems, the H_2Q system produces free radical which makes it not stable during the electrochromic process. The results provide evidence of the bistability source of Urea-N/Rh-M electroacid system.

Supplementary Note 5. Calculation of energy consumption of the bistable billboard

The power consumption of the bistable electrochromic billboard (for 2 days):
$W = UIt = 1.0 \times 0.02 \times 10 \times 4\ J = 0.8\ J$
The power consumption of the LCD (150 W/m^2):
$W = Pt = 150 \times 0.01 \times 2 \times 24 \times 3600\ J = 259\ 200\ J$
The power consumption of the LED (100 W/m^2):
$W = Pt = 100 \times 0.01 \times 2 \times 24 \times 3600\ J = 172\ 800\ J$
The scale: $\eta = 0.8/172\ 800 = 4.63 \times 10^{-6}$

Supplementary Method 1. Materials and Methods

Materials

N, *N*-dimethylbenzene-1,4-diamine, 1-isocyanato-4-methylbenzene, Rhodamine B, phosphorus oxychloride, 1,2-dichloroethane, 4-(dimethylamino)phenyl isocyanate, *N*-methyl-p-toluidine, triethylamine, acetyl chloride, hydroxylammonium chloride, poly(methyl methacrylate) (PMMA), 1-butyl-3-methylimidazolium hexafluorophosphate ([BMIM]PF_6), p-benzoquinone (BQ), and hydroquinone (H_2Q) were purchased from Aladdin.

Transparent indium tin oxide-glass electrode (ITO) and flexible PET (polyethylene terephthalate)-ITO (indium tin oxide) electrode (PET-ITO) were purchased from CSG Holding Co., Ltd. Nafion® NRE-212 membrane (0.05 mm thick, ⩾ 0.92 meq/g exchange capacity) was purchased from Alfa Aesar.

Instrument characterization

UV-vis absorption spectra were measured using a Shimadzu UV-2550 double-beam spectrophotometer. Fluorescence spectra were obtained with a Shimadzu spectrofluorimeter RF-5301PC. Cyclic voltammograms were obtained from Bio-logic electrochemical work station. Solution experiments were performed under argon atmosphere. ESI-HRMS analysis was performed on an Agilent 1290-micrOTOF-Q II mass spectrometer. Accurate masses were reported for the molecular ion $[M+H]^+$ or $[M]^+$. Nuclear magnetic resonance spectra (^{1}H NMR and ^{13}C NMR) were recorded with Varian Mercury (300 MHz) and Bruker 500 MHz NMR spectrometer. IR spectra studies were performed on Vertex 80/80V FT-IR spectrometer with LN-MCT Mid DC detector over the range of 4000-800 cm^{-1} using a KBr plate. Photo-etching was realized by layer mask machine FB30-Z HPWU0300-SKS. Fiber optical spectra were measured on a Maya 2000PRO fiber optical spectrometer with Ocean DH-2000-BAL UV-Vis-NIR lightsource.

The theoretical calculations

All the calculations were performed using the Gaussian 09 program. All molecules were fully optimized using the hybrid M06 functional with 6-311g(d,p) basis set[3]. Then the vibrational spectrum of each molecule has been calculated at the same level of theory to ensure that all structures correspond to true minima of the potential energy surface.

Supplementary Method 2. Synthesis of molecules

3',6'-bis(diethylamino)-2-hydroxyspiro[isoindoline-1,9'-xanthen]-3-one: Rhodamine B (10 mmol) was dissolved in 80 mL 1,2-dichloroethane, then added dropwise to 6 mL of phosphorus oxychloride. The reaction was refluxed for 4 h. Then, the solvent of the reaction mixture was distilled off to obtain violet oil. The residue was dissolved in acetontrile (100 mL), then added dropwise to the solution of hydroxylamine hydrochloride (7 g) and triethylamine (24 mL) in THF (100 mL)/water (20 mL) at ice bath. The reaction was stirred for 4 h at room temperature. The solvent of the reaction mixture was distilled off after the reaction. Then, water was added to the oil, and the suspended solution was filtered. The precipitate was washed several times with water and dried in air, affording a violet‑red powder, 78% yield. ^{1}H NMR (300 MHz, $CDCl_3$) δ = 7.83 (d, *J*=6.1, 1H), 7.43 (p, *J*=7.5, 2H), 7.08 (d, *J*=6.9, 1H), 6.53 (d, *J*=8.6, 2H), 6.44 (s, 2H), 6.30 (d, *J*=8.4, 2H), 3.35 (q, *J*=7.0, 8H), 1.17 (t, *J*=6.4, 12H); ^{13}C NMR (DMSO-d_6, 75 MHz) δ37.962, 41.374, 114.016, 123.611, 127.934, 128.299, 130.517, 130.937, 145.053, 148.699, 156.379

3-(4-(dimethylamino)phenyl)-1-methyl-1-(p-tolyl)urea (Urea-Me): 4-(dimethylamino)phenyl isocyanate (10 mmol) was dissolved in 20 mL acetontrile, then the solution was added dropwised to a solution of 10 mmol of *N*-methyl-p-toluidine also dissolved in 40 mL acetontrile at 0 ^{o}C. The reaction was stirred for 5 h under N_2 atmosphere. The solvent of the reaction mixture was distilled off after the reaction, and the residue was purified by column chromatography to afford a light gray solid, 80% yield. (Supplementary Figure 26) ^{1}H NMR (500 MHz, DMSO-d_6) δ 7.61 (s, 1H), 7.22 – 7.18 (m, 6H), 6.62 (d, J = 8.7 Hz, 2H), 3.20 (s, 3H), 2.81 (s, 6H), 2.31 (s, 3H). ^{13}C NMR (126 MHz, DMSO-d_6) δ 155.04, 146.60, 141.62, 134.90, 129.80, 129.74, 126.22, 121.92, 112.61, 40.67, 37.43, 20.55. LC-HRMS: m/z calc. for $C_{17}H_{22}N_3O$ 284.1757, found 284.1753.

Supplementary Method 3. Preparation of the electrochromic devices

Preparation of the liquid electrochromic device

Electrochromic solution: A mixture of Urea-N (2.7 mg), Rh-M (2.5 mg) and BQ (2.16 mg) in 10 mL [BMIM]PF_6 was stirred for 24 h.

The electrochromic device was prepared by two ITO electrodes and a spacer (thickness: 0.1 mm). Then, the electrochromic solution was injected into electrochromic device as Supplementary Figure 5a.

Preparation of the photo-etched electrodes

ITO glass electrodes or PET-ITO electrodes are photo-etched by layer mask machine FB30-Z HPWU0300-SKS.

The the solid electrochromic devices with photo-etched ITO electrodes were fabricated according to the procedure of the preparation of solid electrochromic devices.

Preparation of the flexible solid electrochromic device

Firstly, the transparent counter film layer was deposited by spin coating on the first PET-ITO. Next, the transparent conductive film was deposited on the top of the counter film. The transparent colorless electrochromic film was deposited by spin coating on the second PET-ITO. Finally, the two PET-ITO are assembled together.

Preparation of the smart glasses device

Firstly, the transparent counter film layer was deposited by spin coating on the first glasses-shape ITO. Next, the transparent conductive film was deposited on the top of the counter film. The transparent colorless electrochromic film was deposited by spin coating on the second glasses-shape ITO. Finally, the two glasses-shape ITO are assembled together.

Applications of the multicolor bistable electrochromic devices

In order to achieve multicolor display in our electroacid system, another three pH sensitive dyes, *N*,*N*-dimethyl-4-(2-pyridin-4-ylethenyl)aniline (**ASY**), 6'-(diethylamino)-2'-(phenylamino)spiro[isobenzofuran-1(3H),9'-[9H]xanthene]-3-one (**TFG**), and 2-anilino-6-dibutylamino-3-methylfluoran (**ODB-2**), were selected to build up the solid electrochromic devices instead of Rh-M separately.

Preparation of the multicolor bistable electrochromic device with photo-etched electrode

Firstly, the transparent counter film layer was deposited by spin coating on the first glasses-shape ITO. Next, the transparent conductive film was deposited on the top of the counter film. The transparent electrochromic films with different acid sensitive molecules were deposited on the corresponding parts of the etched ITO electrode. Finally, the two ITO electrodes are assembled together.

Preparation of Li-Nafion membrane solid bistable device

Composition of solution of each layer is as follow:

Electrochromic solution: A mixture of PMMA (2.4 g), [BMIM]PF_6 (2.6 mL), **Urea-N** (81 mg), **Rh-M** (150 mg), and BQ (110 mg) in 20 mL acetonitrile was stirred for 24 h.

Counter solution: A mixture of PMMA (2.4 g), [BMIM]PF_6 (2.6 mL), BQ (220 mg) and H_2Q (440 mg) in 20 mL acetonitrile was stirred for 24 h.

Solution of electrochromic layer was coated on an ITO glass electrode, and solution of counter layer was coated on another ITO glass electrode. After the vaporization of the solvent, uniform films were attached to the two electrodes. Li-Nafion membrane was then coated onto the film of counter layer. The electrodes with films were put together and the film was sandwiched between the electrodes to fabricate the solid devices.

Supplementary References

[1] Gupta, N., & Linschitz, H. Hydrogen-bonding and protonation effects in electrochemistry of quinones in aprotic solvents. *J. Am. Chem. Soc.* **119**, 6384-6391 (1997).

[2] Astudillo, P. D., Tiburcio, J., & González, F. J. The role of acids and bases on the electrochemical oxidation of hydroquinone: Hydrogen bonding interactions in acetonitrile. *J. Electroanal. Chem.* **604**, 57-64 (2007).

[3] Frisch, M. J. et al. Gaussian 09, Revision A.02; Gaussian, Inc.: Wallingford, CT (2009).
