# Supplementary material for: Bio-inspired ultra-high energy efficiency bistable electronic billboard and reader
Source: Nat Commun. 2019 Apr 5;10:1559. doi: 10.1038/s41467-019-09556-5 (PMC6450890; doi:10.1038/s41467-019-09556-5)
Supplement: Supplementary file 2 — Description of Additional Supplementary Files [file 41467_2019_9556_MOESM2_ESM.pdf]

Description of Additional Supplementary Files

File Name: Supplementary Movie 1
Description: Bistable Information Display

File Name: Supplementary Movie 2
Description: Bistable and Fast Display

File Name: Supplementary Movie 3
Description: Bistable Pixel Information Display

File Name: Supplementary Movie 4
Description: Bistable Pixel Display

File Name: Supplementary Movie 5
Description: Bistable Flexible Display

File Name: Supplementary Movie 6
Description: Bistable Wearable Glasses
